# Highly Selective Electroreduction of CO_2_ to CH_4_ on Cu–Pd Alloy Catalyst: the Role of Palladium‐Adsorbed Hydrogen Species and Blocking Effect

**DOI:** 10.1002/advs.202417247

**Published:** 2025-03-26

**Authors:** Jinyan Huang, Ye Yang, Xuexue Liang, Bing Chen, Yue Shen, Yan Chen, Jielian Yang, Yinglin Yu, Fang Huang, Huibing He, Peican Chen, Liya Zhou, Anxiang Guan

**Affiliations:** ^1^ School of Chemistry and Chemical Engineering Guangxi Key Laboratory of Electrochemical Energy Materials State Key Laboratory of Featured Metal Materials and Life‐cycle Safety for Composite Structures Guangxi Key Laboratory of Petrochemical Resource Processing and Process Intensification Technology Guangxi Colleges and Universities Key Laboratory of Applied Chemistry Technology and Resource Development Guangxi University Nanning 530004 China

**Keywords:** *H adsorption, blocking effect, CO_2_ electroreduction, hydrogenation

## Abstract

Electroreduction of CO_2_ to chemical fuels offers a promising strategy for controlling the global carbon balance and addressing the need for the storage of intermittent renewable energy. In this work, it is demonstrated that tuning adjacent active sites enables the selection of different reaction pathways for generating C_1_ or C_2_ products during the electroreduction of CO_2_. Cu and Cu–Pd alloy catalysts with different atomic ratios are synthesized and investigated to elucidate their different electroreduction selectivities for CO_2_ electroreduction. Cu catalyst favors the formation of C_2_ products since the neighboring active Cu sites are beneficial for coupling adjacently adsorbed ^*^CO and ^*^CHO intermediates. Cu alloyed with Pd introduces a blocking effect and increases the intermolecular distance between adjacent adsorbed ^*^CO and ^*^CHO intermediates. Therefore the selectivity for the C_2_H_4_ pathway decreas while the CH_4_ pathway is enhanced. Moreover, the existence of adsorbed ^*^H species on Pd atoms also played a significant role in boosting CO_2_ electroreduction to CH_4_ by facilitating the hydrogenation of ^*^CO intermediates. This work reveals the key role of ^*^H species adsorbed on Pd atoms and the blocking effect between active sites for CH_4_ formation, which is helpful for the design of copper‐based catalysts for desired products.

## Graphical abstract

The blocking effect induced by the Pd atom would interrupt the C─C coupling reaction by increasing the intermolecular distance between adjacent ^*^CO and ^*^CHO intermediates adsorbed on the Cu atoms when introduced Pd into the Cu lattices and the ^*^CO protonation effect of palladium‐adsorbed ^*^H species boosts the CH_4_ formation on the Cu–Pd alloy catalyst during the CO_2_ electroreduction.

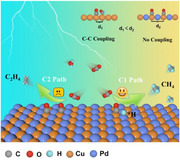

## 1 Introduction

As the consumption of fossil fuels continues to increase, the emission of the greenhouse gas carbon dioxide (CO₂) has been aggravated, resulting in severe environmental and climate issues and extensive research on novel energy storage and conversion devices.^[^ ^1^, ^2^, ^3^ ^]^ The electrocatalytic CO₂ reduction reaction (CO₂RR) is a prospective strategy to alleviate the above problems by transforming CO₂ into high‐value‐added chemicals and fuels.^[^ ^4^, ^5^, ^6^ ^]^ Specifically, the electrocatalytic conversion of CO₂ into methane (CH₄) is an appealing approach. CH₄ is a common raw material for natural gas and a substitute for gasoline, having a combustion‐specific heat as high as 73.39 kJ mol⁻¹ and thus possessing high economic benefits.^[^ ^7^, ^8^ ^]^ Nevertheless, CH₄ is the C_1_ product with the highest degree of reduction, which requires the transfer of 8 electrons, and competes with carbon monoxide (CO) which only requires the transfer of two electrons.^[^ ^9^, ^10^, ^11^ ^]^ Therefore, exploring the electrocatalytic reduction process of CO₂ to CH₄ is of great significance for a deeper understanding of the characteristics of CO₂RR.

Copper (Cu)‐based catalysts are currently the sole materials that can convert CO₂ into various hydrocarbons and alcohols.^[^ ^12^, ^13^ ^]^ According to previous reports, Cu alloys,^[^ ^14^ ^]^ oxide‐derived compounds,^[^ ^15^ ^]^ and Cu single‐atom.^[^ ^16^ ^]^ catalysts can selectively generate target products such as CO,^[^ ^17^ ^]^ formate (HCOO⁻),^[^ ^18^ ^]^ CH₄,^[^ ^19^ ^]^ ethylene (C₂H₄),^[^ ^20^ ^]^ and ethanol (CH₃CH₂OH).^[^ ^21^ ^]^ The design of Cu alloys has been demonstrated to be an effective approach for regulating the products of the CO₂ reduction reaction, including the type and content of added metals.^[^ ^5^, ^12^, ^14^ ^]^ This is because the alloy can effectively control the binding strength with intermediates by further optimizing the surface charge distribution and adjusting the active sites that provide products, thereby accelerating CO₂RR and enhancing the reduction selectivity of CO₂.^[^ ^22^ ^]^ It is generally accepted that the intermediate ^*^CO (derived from ^*^COOH), which is formed by the activation of CO₂ and H₂O on Cu‐based catalysts, plays a significant role in the formation of CO₂ deep reduction products.^[^ ^23^ ^]^ This understanding allows for the design of specific Cu catalysts to control subsequent hydrogenation or C─C coupling to produce desired products with high selectivity, such as CH₄ and C₂H₄. Therefore, understanding the interaction between the key ^*^CO intermediates and the catalyst surface is essential for controlling the CO₂RR process.

Here we designed Cu–Pd alloys with various compositions as an electrocatalyst for methane synthesis from CO_2_RR. It could be seen that C_2_H_4_ products were more likely to be generated on the Cu catalyst without Pd alloying, while CH_4_ is preferentially generated over C_2_H_4_ on the Cu–Pd alloy, displaying significant differences in CO_2_RR processes between Cu and Cu–Pd alloy. For Cu without Pd doping catalyst, ^*^CO and ^*^CHO species have more opportunities to bind with each other on the Cu surface, promoting the conversion process of C─C coupling reaction, enhancing the reaction pathway of C_2_ and thus generating C_2_H_4_ product. However, the addition of Pd introduced a blocking effect and increased ^*^H coverage, promoting the hydrogenation process of ^*^CO and enhancing the CH_4_ formation. The mechanism for the high methane selectivity on the Cu–Pd alloys was investigated by a series of experiments and calculations.

## 2 Results and Discussion

**Figure 1:**
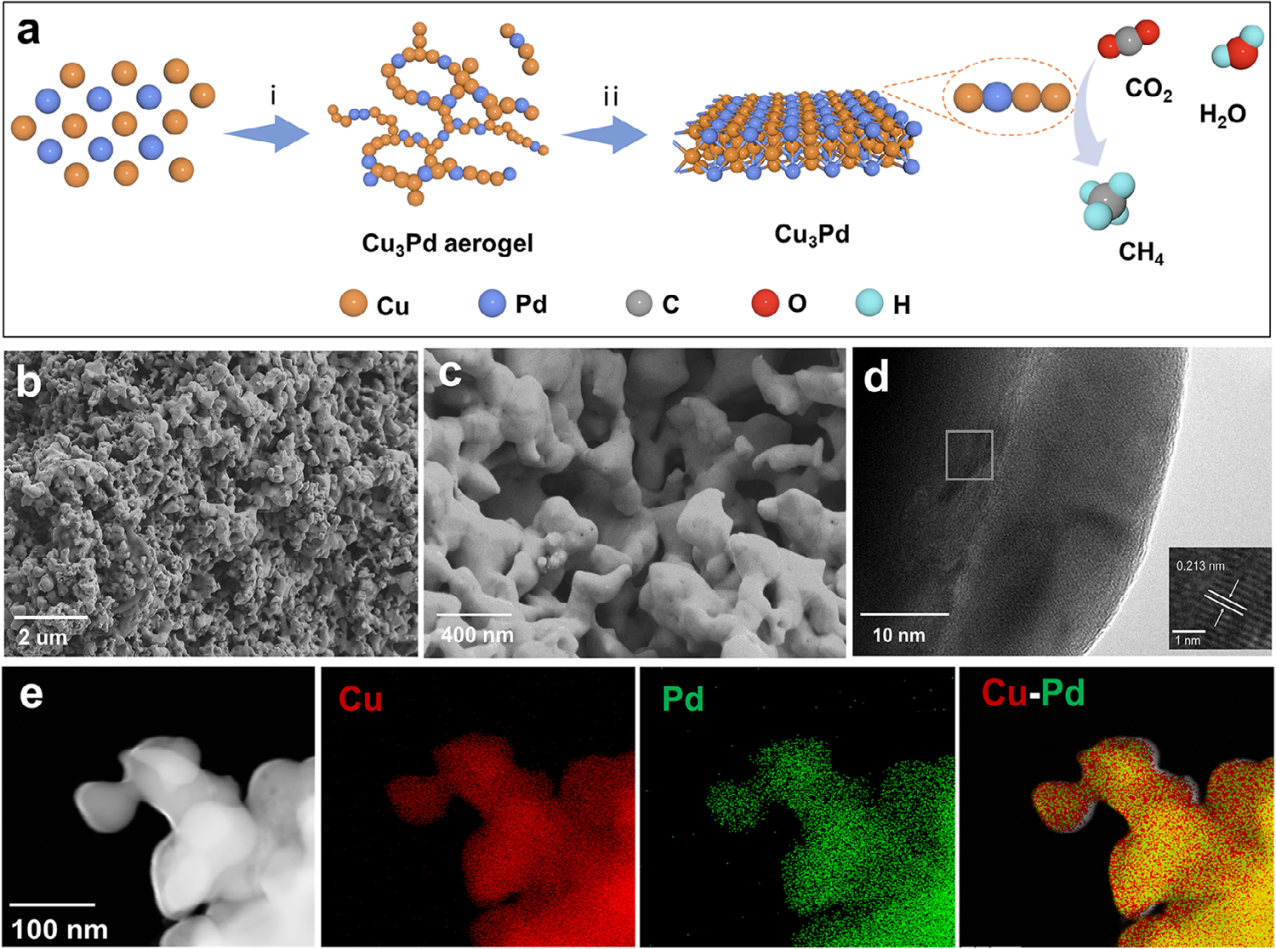
a) Schematic illustration of the synthetic procedure for Cu–Pd alloys. b) Low‐ and c) high–resolution SEM images of Cu_3_Pd. d) High‐resolution TEM images of Cu_3_Pd. e) EDS elemental maps of the Cu_3_Pd alloy catalyst.

The preparation of Cu–Pd alloys with diverse stoichiometric Cu/Pd ratios was synthesized via a reduction approach, which was schematically illustrated in **Figure** **1**a Scanning electron microscopy (SEM) and transmission electron microscopy (TEM) images were employed to characterize the morphology of the samples. Both the Cu and Cu–Pd samples exhibited an interconnected network structure with nanoscale pores of different sizes (Figure 1b,c; Figures S1–S3, Supporting Information). High‐resolution transmission electron microscopy (HRTEM) images revealed that the Cu₃Pd sample shows a lattice spacing measured as 0.213 nm, corresponding to the Cu₃Pd (111) plane (Figure 1d; Figure S4, Supporting Information). The TEM images of Cu and CuPd samples were also displayed in Figures S5–S6 (Supporting Information) for comparison, which displayed their intrinsic lattice spacings. Energy‐dispersive X‐ray spectroscopy (EDS) was used to disclose the distribution of Cu and Pd atoms in the catalyst. Clearly, Cu and Pd elements showed a uniform distribution all around the samples (Figure 1e; Figure S7, Supporting Information).

**Figure 2:**
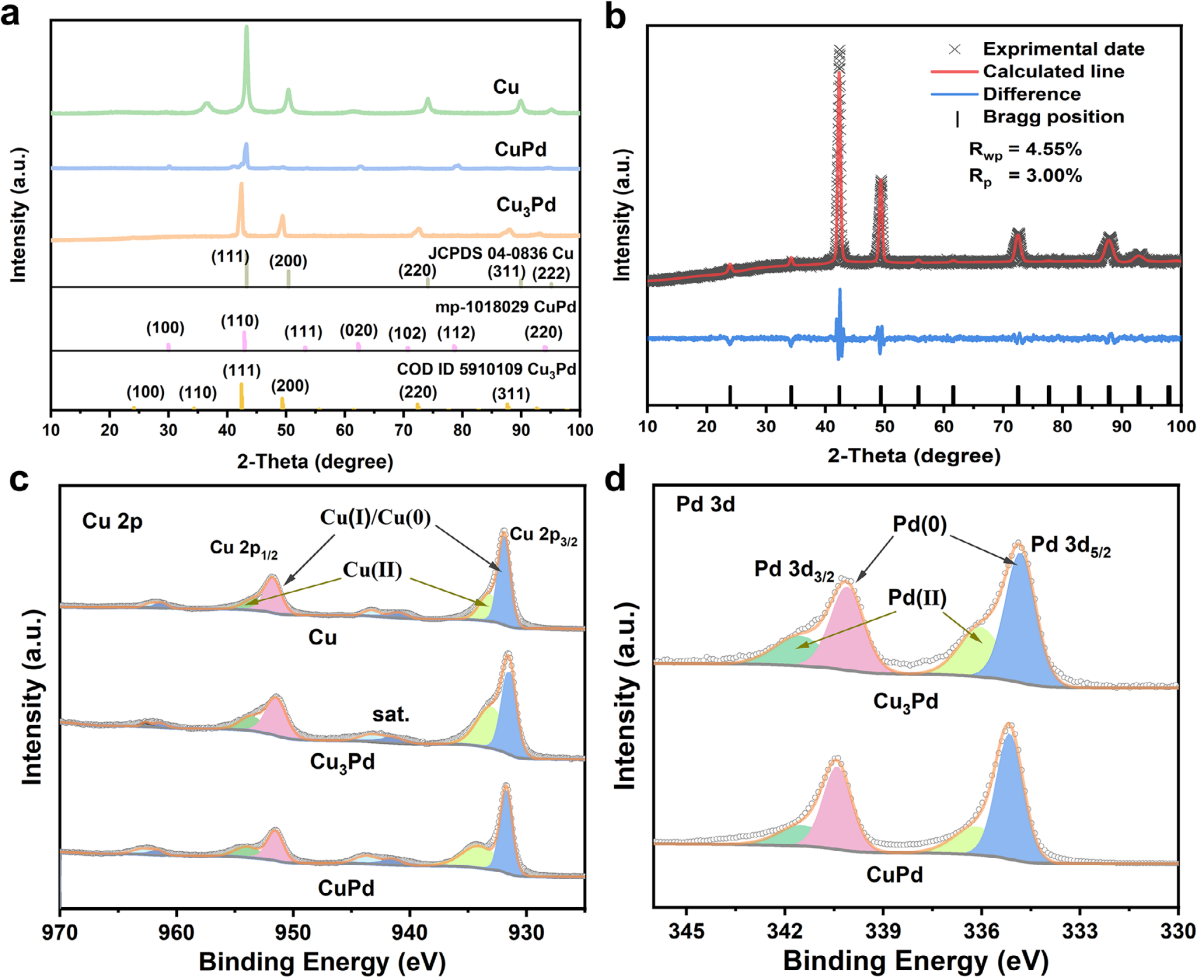
a) XRD patterns of Cu, Cu_3_Pd and CuPd alloy catalysts. b) Calculated profile based on Rietveld refinement for Cu_3_Pd alloy. c) Cu 2p XPS spectra of Cu and different Cu–Pd alloys. d) Pd 3d XPS spectra of different Cu–Pd alloys.

The powder X‐ray diffraction (XRD) patterns indicated that the catalysts were successfully synthesized (**Figure** **2**a). For Cu_3_Pd sample, its XRD pattern showed four distinct diffraction peaks at 42.40°, 49.41°, 72.53°, and 87.98°, corresponding to the (111), (200), (220), and (311) crystal planes of a face‐centered‐cubic (fcc) Cu_3_Pd phase. Further verification of the crystal phase of the Cu_3_Pd sample was carried out through Rietveld refinement analysis (Figure 2b; Table S1, Supporting Information), and the crystal structure of the Cu_3_Pd alloy fitted well with an ordered cubic structure, suggesting that Cu and Pd atoms were alternately arranged at neighboring sites in the fcc‐based lattice. The ICP measurement results of Cu_3_Pd and CuPd samples showed that all samples were successfully stoichiometrically synthesized (Table S2, Supporting Information). The chemical states of Cu and Pd were analyzed by X‐ray photoelectron spectroscopy (XPS). In the Cu 2p spectra of the Cu catalyst (Figure 2c), two main peaks centered at 931.7 and 951.7 eV confirmed the existence of Cu(I) or Cu(0) species, along with their corresponding satellite peaks located at ≈941.1 and 961.4 eV.^[^ ^24^, ^25^ ^]^ The peaks at 933.3 and 953.7 eV, and the satellite peaks at 943.4 and 962.4 eV, were ascribed to Cu(II) species with an unfilled electron state in the Cu 3d^9^ orbital.^[^ ^24^, ^26^, ^27^ ^]^ Since the binding energy values of Cu(0) and Cu(I) are similar, it is extremely difficult to distinguish them from the XPS spectra based merely on the Cu 2p data. Therefore, the X‐ray‐generated Auger Cu LMM spectra were obtained for a more reliable identification of Cu(0) and Cu(I). As depicted in Figure S8 (Supporting Information), the Cu LMM Auger spectra of all samples showed two peaks at 916.6 and 918.4 eV, indicating the coexistence of Cu(I) and Cu(0) species.^[^ ^4^, ^28^ ^]^ Besides, it can be observed that the content of the Cu(I) component is higher than that of the Cu(0) component in the Cu sample, a situation contrary to that in the Cu_3_Pd and CuPd samples. This disparity is likely due to the alloying layer present in the Cu–Pd alloy samples, which acts as a protective barrier, shielding Cu from oxidation. The formation of Cu(I) and Cu(II) species can be attributed to the spontaneous oxidation of the Cu nanoparticle surface when it is exposed to air. Figure 2d displays the Pd 3d XPS spectra of the Cu–Pd alloy samples. Two prominent peaks at binding energies of ≈335.2 and 340.4 eV were observed, which correspond to the Pd 3d_5/2_ and 3d_3/2_ electrons of metallic Pd, respectively. Peak deconvolution analysis revealed two distinct doublets in the Pd 3d region. The dominant doublet centered at 335.2/340.4 eV is characteristic of metallic Pd(0), while the weaker doublet (336.4/341.7 eV) can be attributed to oxidized Pd(II) species such as PdO. Collectively, these results demonstrate that the majority of Pd in the Cu–Pd alloy samples exists in the metallic oxidation state Pd(0).^[^ ^29^, ^30^ ^]^

**Figure 3:**
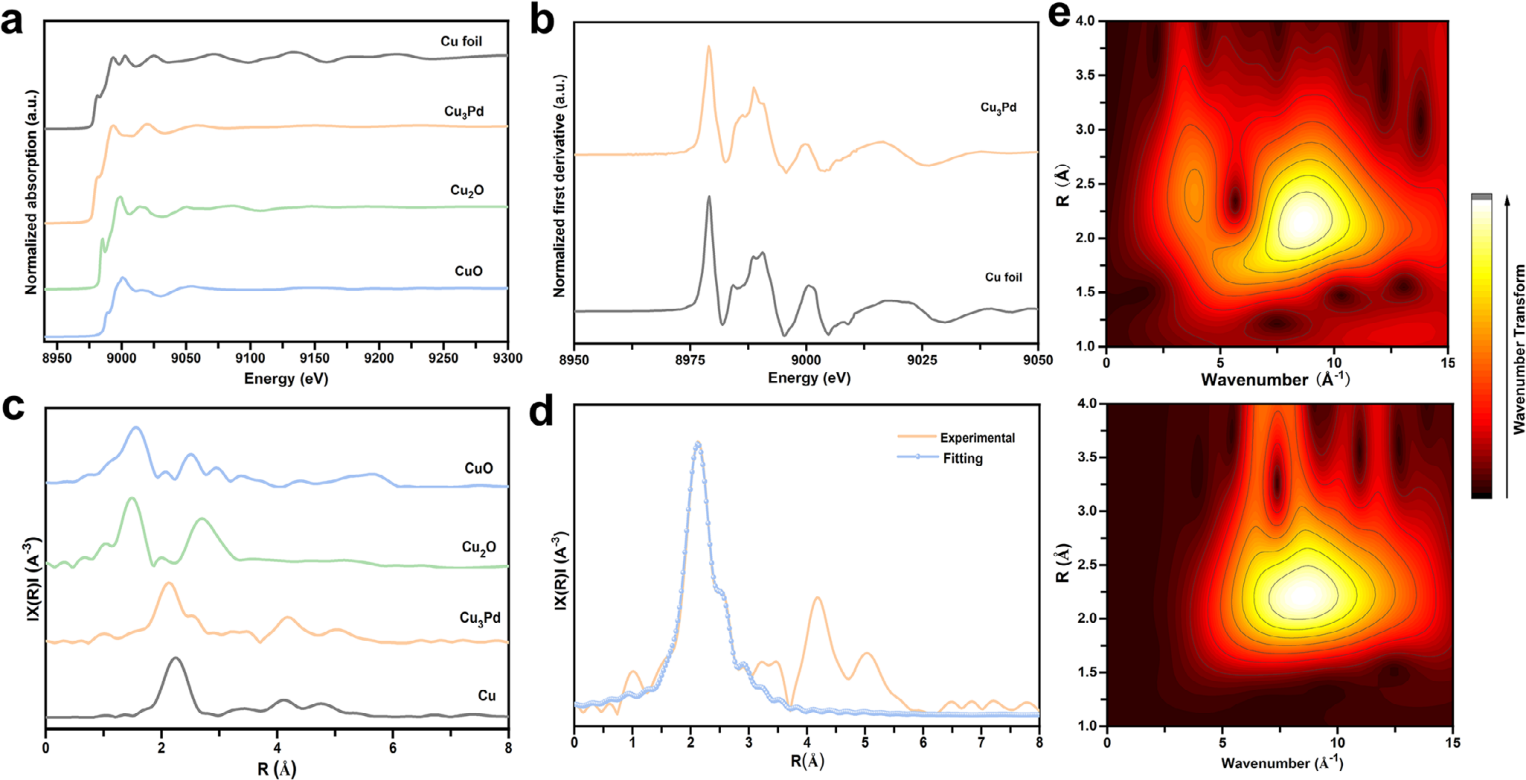
a) Cu K‐edge XANES spectra of Cu foil, Cu_3_Pd, Cu_2_O, and Cu. b) first derivative spectra derived from the XANES region. c) FT‐EXAFS of Cu_3_Pd with reference to Cu foil, Cu_2_O, and CuO. d) EXAFS fitting curve for Cu_3_Pd at the Cu K edge. e)Wavelet transform images of EXAFS data at Cu K‐edge with the optimized Morlet parameter for Cu_3_Pd (lower panel for Cu, upper panel for Cu_3_Pd).

In order to gain a deeper understanding of the chemical properties and structure of Cu and Pd atoms in Cu_3_Pd, we carried out X‐ray absorption near edge structure (XANES) and extended X‐ray absorption fine structure (EXAFS) measurements. The absorption edge of Cu_3_Pd at the Cu K‐edge was similar to that of Cu foil (as shown in **Figure** **3**a), and the characteristic peak of Cu_3_Pd in the first derivative spectrum positioned at the same energy region compared to that of Cu foil (Figure 3b), which verified that the oxidation state of Cu in the Cu_3_Pd sample was zero. The same results could be obtained from Figure S9a (Supporting Information) for Pd in the Cu_3_Pd sample by comparing it to Pd foil. The EXAFS spectra of Cu_3_Pd at the Cu K‐edge exhibited only a dominant peak positioned at 2.13 Å (Figure 3c), which was similar to that of Cu foil and was attributed to the Cu─Cu scattering path. The corresponding extended X‐ray absorption fine structure (EXAFS) fittings revealed bond lengths of ≈2.23, 2.11, 2.36 and 2.01Å for Cu─Cu, Cu–Pd, Pd–Cu and Pd–Pd bonds, respectively (Figure 3d; Figures S9b‐d and S10 and Table S3, Supporting Information), and the corresponding coordination numbers were ≈2.58 (Cu─Cu), 8.65 (Cu─Pd), 8.16 (Pd─Cu) and 2.98 (Pd─Pd) (Table S3, Supporting Information). Additionally, the wavelet transforms (WT) analysis and the WT contour plots for the Cu k‐edge showed only one intensity maximum at 8.76 Å^−1^ for Cu_3_Pd (Figure 3e), corresponding to a radial distance of 2.13 Å and further indicating the existence of Cu(0) species. The wavelet transform (WT) analysis and the WT contour plots for the Pd k‐edge were also displayed in Figure S11 (Supporting Information), which indicated the Pd(0) species were present in the catalyst.

**Figure 4:**
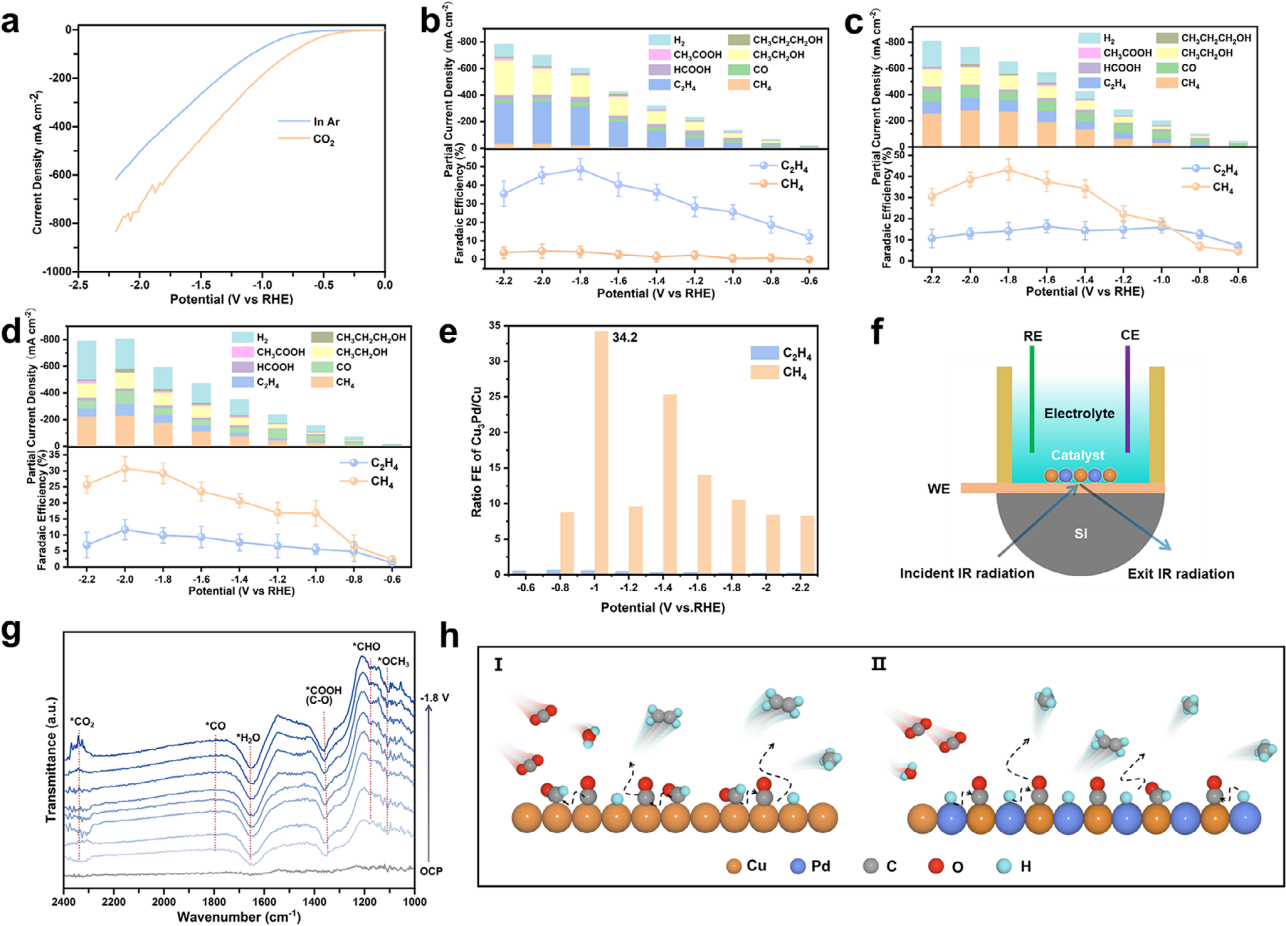
a) LSV curves for the Cu_3_Pd catalyst measured in CO_2_‐flowed KOH electrolyte and in Ar‐flowed KOH electrolyte. CO_2_ electroreduction performances of b) Cu catalyst, c) Cu_3_Pd, and d) CuPd catalyst. e) The ratios of FE_C2H4_ and FE_CH4_ between Cu_3_Pd and Cu samples. f) Schematic diagram of operando ATR‐SEIRAS experimental setup. WE, working electrode, CE, counter electrode, RE, reference Electrode. g) Operando ATR‐SEIRAS spectra of Cu_3_Pd catalyst for CO_2_ electroreduction at different potentials in CO_2_‐saturated 0.5 m KHCO_3_ electrolyte. h) Schematic illustrations for the mechanism of CH_4_ formation on Cu and Cu_3_Pd catalysts.

The electrochemical CO_2_RR performance of Cu and Cu–Pd alloy catalysts was then evaluated in a flow cell with a three‐electrode configuration (Figure S12, Supporting Information). A 1 m potassium hydroxide (KOH, pH 14) aqueous solution was used as the electrolyte, and the total mass of the loaded catalysts was 1.0 mg cm^−2^. All the measured potentials in this work were relative to an Ag/ AgCl^−1^ (3M KCl) reference electrode, and all the reported voltages were converted to the reversible hydrogen electrode (RHE) scale. The linear sweep voltammetry (LSV) measurement of Cu_3_Pd with the CO_2_ flow exhibited larger current densities than with the Ar flow (**Figure** **4**a), implying the occurrence of CO_2_RR. The LSV curves of Cu, Cu_3_Pd, and CuPd catalysts displayed similar levels of current densities, indicating their excellent CO_2_RR electrocatalytic properties (Figure S13, Supporting Information). The electrocatalytic CO₂RR products collected from −0.6 to −2.2 V vs. RHE RHE were detected and analyzed by online gas chromatography (GC) and nuclear magnetic resonance hydrogen spectroscopy (¹H‐NMR) (Figure S14,S15, Supporting Information). The Cu catalyst exhibited good catalytic performance in CO₂ reduction, with C_2_H_4_ as the main reduction product. At a potential of −1.8 V vs. RHE, the Faradaic efficiency of C₂H₄ (FE_C₂H₄_) could reach as high as 48.67%, superior to the CH_4_ formation with a maximum FE_CH4_ of 4.56% at the potential of −2.0 V versus RHE. The corresponding partial current densities for the maximum C_2_H_4_ and CH_4_ formation were −286.80 and −31.70 mA cm^−2^ at −1.8 and −2.0V vs. RHE (Figure 4b), respectively.

In comparison with the pure Cu catalyst, CH₄ was manifestly generated in the reduction products for Cu–Pd alloy electrocatalysts, whilst the selectivity of C₂H₄ progressively decreased. As observed, the pure Cu catalyst exhibited the highest Faradaic efficiency of C₂H₄ production (FE_C₂H₄_) of 48.67% and the best C₂H₄ selectivity of 53.14% (defined as the ratio between C₂H₄ and all CO_2_RR products) (Figure S16, Supporting Information) at −1.8 V vs. RHE among all the samples. While the Cu₃Pd catalyst exhibited the highest Faradaic efficiency of CH₄ production (FE_CH₄_) of 43.23% and the best CH₄ selectivity of 48.16% at −1.8 V vs. RHE (Figure 4c; Figure S17, Supporting Information), corresponding to a partial current density for CH_4_ production of −269.68 mA·cm^−2^. Similarly, the CuPd catalyst showed a FE_CH₄_ of 29.24% at −1.8 V vs. RHE, corresponding to the CH₄ selectivity of 40.82% among all samples (Figure 4d; Figure S17, Supporting Information). Figure 4e shows the ratios of CH_4_ between Cu and Cu_3_Pd catalysts from −0.6 to −2.2 V vs. RHE. As shown, the ratios of CH_4_ between Cu and Cu_3_Pd catalysts reached a peak value of 34.2, indicating the formation of CH_4_ was more favorable on the Cu_3_Pd catalyst than on Cu catalyst. Contrarily, all the ratios of C_2_H_4_ between Cu_3_Pd and the Cu catalysts were smaller than 1, suggesting the Cu catalyst was inclined to electroreduce CO_2_ to form C_2_H_4_. The same results could be obtained for the comparison between CuPd and Cu, and the corresponding maximum ratio of CH_4_ was 31.6 (Figure S18, Supporting Information). Notably, FE_CH4_ showed a decreased trend with the increase of Pd content in the catalyst (Figure S19, Supporting Information). This is because the Pd atom is inert for CO_2_ reduction, which could be further confirmed by that the main CO_2_RR reduction product of Pd catalyst is H_2_ (Figure S20, Supporting Information), and meanwhile, this also proves that Pd atoms are favorable for the generation of ^*^H species.

To study the reduction mechanism of carbon dioxide on the Cu_3_Pd catalyst, the operando attenuated total reflection‐surface‐enhanced infrared absorption spectroscopy (ATR‐SEIRAS) tests were employed to observe the adsorbed intermediates. In the transmittance versus wavenumber spectrum, negative peaks represent the generation trend of species, while the positive peaks represent the consumption trend. The experimental setup and corresponding schematic diagram are shown in Figure S21 (Supporting Information) and Figure 4f. As shown in Figure 4g, the peaks at 2340 cm^−1^ corresponded to the ^*^CO_2_ adsorption peaks, while the peak of 1640 cm^−1^ was ascribed to the bending vibration of ^*^H_2_O adsorbed on the catalyst surface.^[^ ^31^ ^]^ Meanwhile, the peak of 1360 cm^−1^ with a large intensity was attributed to the C─O bond vibration of the ^*^COOH group, which was an intermediate for the production of CO_2_ deep reduction products.^[^ ^32^ ^]^ The absence of the characteristic peaks of ^*^CO (2058‐2024 cm^−1^) intermediate may be due to the fast hydrogenation process of ^*^CO to form ^*^OCH_3_ (1110 cm^−1^) and ^*^CHO (1178 cm^−1^) species, both of which were key intermediates for CH_4_ formation.^[^ ^32^, ^33^ ^]^ Different from Cu_3_Pd, the ATR‐SEIRAS spectra of Cu showed peaks at 1510 and 1078 cm^−1^ associated with ^*^COCHO species (Figure S22, Supporting Information).^[^ ^34^ ^]^ As the applied potential became increasingly negative, the peak intensity of the ^*^COCHO species gradually diminished, which could be responsible for the enhanced C─C coupling on Cu and thus further confirmed the preferred C_2_ production on Cu.

A mechanism was proposed to explain why the Cu catalyst boosted the reduction of CO_2_ to C_2_H_4_ while CH_4_ formation was enhanced on the Cu–Pd alloy catalysts (Figure 4h). From the perspective of catalyst components, the Cu–Pd alloy catalyst has an additional component Pd compared to the pure Cu catalyst, which may be the main reason for the inconsistent electrocatalytic reduction performance of CO_2_ between the two catalysts. As for C_2_H_4_ formation, ^*^COCHO is recognized as a critical intermediate during the formation of C_2_ (or C_2+_) products in the electroreduction of CO_2_. The pure Cu catalyst is composed of countless adjacent Cu atoms. In theory, every Cu atom can electroreduce CO_2_ to ^*^CO and then couple with one another to generate ^*^COCHO species, as depicted in Figure 4h, panel I. As a consequence, C_2_ products like C_2_H_4_ are far more likely to be produced on the Cu catalyst compared to C_1_ products such as CH_4_. However, in the case of the Cu–Pd alloys, the neighboring Cu atoms were separated by the Pd atoms that were inner for CO_2_ activation. Thus, the presence of Pd atoms exerted a blocking effect on the coupling of intermediate species by increasing the distance between ^*^CO and ^*^CHO species on Cu atoms (Figure 4h, panel II, and Figure S23, Supporting Information). Moreover, Pd atoms could boost the activation of H_2_O to form ^*^H species, thus accelerating the ^*^CO hydrogenation to form ^*^CHO on the Cu–Pd alloy catalysts, followed by a further reduction to CH_4_. It is worth noting that as the Cu/Pd ratio changed from 3:1 to 1:1, the Faradaic efficiency of CH₄ gradually decreased, while the by‐products of H₂ gradually increased. This is because when the Cu/Pd ratio is 3:1, the ^*^H species generated on Pd atoms could rapidly protonate the ^*^CO species on Cu atoms to form ^*^CHO species and finally generate CH₄. However, when the Cu/Pd ratio is further increased to 1:1, the contents of ^*^H species also increase due to the increase of Pd content, and excessive ^*^H species that cannot couple with ^*^CO in a timely manner will couple with each other to form H₂. Therefore, the by‐product H₂ of CuPd is more than that of Cu₃Pd. In conclusion, the blocking effect and the ^*^H species induced by Pd atoms are the main reasons for the Cu–Pd catalyst electroreduce CO_2_ to CH_4_ instead of C_2_H_4_.

**Figure 5:**
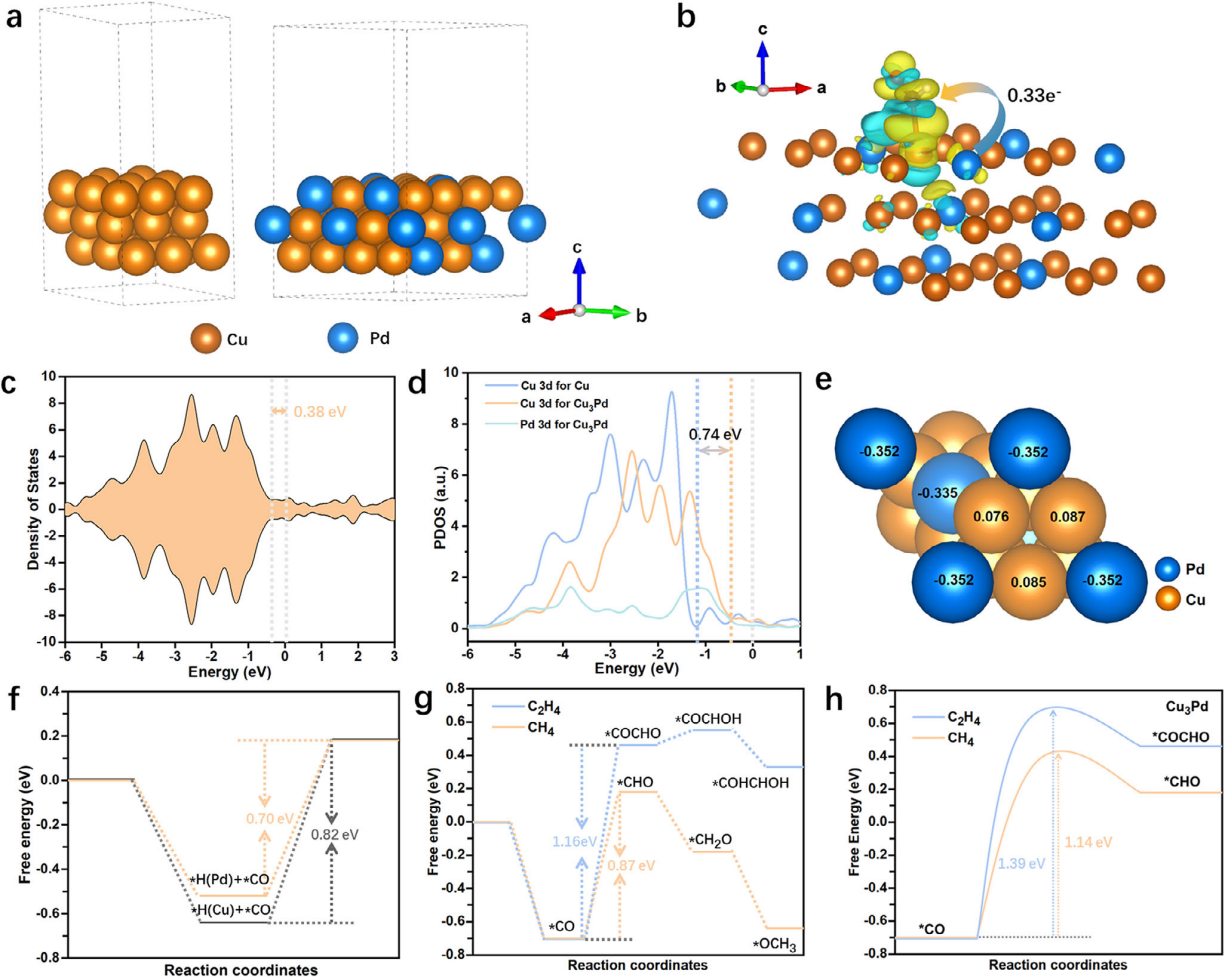
a) Theoretical model of Cu and Cu_3_Pd: the orange, and blue spheres represent Cu and Pd atoms, respectively. b) Differential charge density distributions of Cu_3_Pd with the ^*^CHO intermediates. c) The calculated total density of states for Cu_3_Pd. d) The projected density of states (PDOS) of d orbitals of Cu and Pd atoms on pure Cu and Cu_3_Pd. e) Bader charges analysis of Cu_3_Pd catalyst. Negative values represent a negative charge on the atom. f) The energy contribution of Pd‐adsorbed ^*^H species to ^*^CO hydrogenation compared to that of Cu‐adsorbed ^*^H species. g) Reaction paths and free energy diagrams of CO_2_ reduction to CH_4_ and C_2_H_4_ on Cu_3_Pd catalysts. h) The activation energy calculations for the conversion of ^*^CO to ^*^CHO and ^*^COCHO on Cu_3_Pd.

To gain more insights into the mechanism for CH_4_ generation on the Cu–Pd alloy catalysts, the Gibbs free energy diagrams of CO_2_ reduction to CH_4_ over Cu and Cu_3_Pd catalysts were calculated based on density functional theory (DFT). For the calculations, we adopted a face‐centered cubic (fcc) model for the Cu and Cu₃Pd samples based on the XRD results and the fcc structure has the lowest formation energy among the various phases.^[^ ^35^ ^]^ When constructing our models, we incorporated the Cu (100) and Cu₃Pd (111) facets, which have been identified as being active in the CO₂ reduction reaction,^[^ ^36^, ^37^ ^]^ and the Cu and Cu_3_Pd models were constructed as shown by **Figure** **5**a. The differential charge density distributions analysis (Figure 5b; Figure S24, Supporting Information) indicates stronger electron interaction with ^*^CHO species in Cu_3_Pd than Cu (i.e., 0.33e^−^ vs. 0.27e^−^). On the contrary, the Cu catalyst has a stronger electronic interaction with the ^*^COCHO species compared to Cu_3_Pd catalyst (i.e., 0.18e^−^ vs. 0.14e^−^) in Figure S25 (Supporting Information), indicating that Cu is more conducive to electroreduce CO_2_ into C_2_ products (such as C_2_H_4_). However, the addition of Pd to Cu is unfavorable for the formation of C_2_ products.

The total density of states (TDOS) of Cu and Cu_3_Pd were displayed in Figures 5c and Figure S26 (Supporting Information). It was observed that there were more electronic states near the Fermi level for Cu_3_Pd than Cu; this was because the d‐orbital of Pd hybridized with the d‐orbital of Cu, thus facilitating electron transfer due to their different electronegativities. As a result, the Cu_3_Pd possessed a stronger intrinsic conductivity and electron migration ability compared to Cu, implying a reduced filling of antibonding orbitals of Cu_3_Pd to give a strong interaction with the reactants.^[^ ^38^ ^]^ The projected density of states (PDOS) of d orbitals of Cu and Pd atoms were further calculated, and the results showed an obvious right shift of PDOS of Cu d orbitals in surface alloy compared to pure Cu, and the corresponding d band center is thus nearer to the Fermi level than that on pure Cu by 0.74 eV (Figure 5d). As a result, the bonding of adsorbed species is all strengthened on the Cu_3_Pd compared to the Cu. As we all know, the stronger the adsorption ability for adsorbates, the easier it is for species to couple with each other during CO_2_ electroreduction. However, judging from the results of the CO₂ electroreduction experiments, the Cu₃Pd catalyst, which has a relatively strong ability to adsorb intermediate species, is unfavorable for the formation of C─C coupling products (such as C_2_H_4_) and is more inclined to form CH₄ products. This is because the blocking effect mentioned above and the ^*^H species adsorbed on Pd atoms play a significant role in the catalytic reaction pathway. It could be concluded that geometric/structural effects are likely to play a more significant role than electronic effects in determining the catalytic selectivity and activity, which is similar to Kenis's viewpoint.^[^ ^39^ ^]^

Bader charge analysis was conducted to study the charge distribution on the Cu₃Pd surface, aiming to reveal the electronic effects that might account for the observed selectivity trend of the CO₂ reduction reaction. It was found that in the Cu₃Pd alloys, Cu donates charge to Pd, resulting in Pd being negatively charged. As illustrated in Figure 5e, on the Cu₃Pd (111) facet, the Bader charge of Cu is +0.076/0.085/0.087|e|, whereas that of Pd is −0.335/−0.352|e|. This result is consistent with the electronegativity trend on the Pauling electronegativity scale, where the electronegativity of Pd (2.2) is greater than that of Cu (1.9). According to the principle of attraction between positive and negative charges, the electronegative Pd atom is more favorable for adsorbing H⁺ to form the ^*^H species compared with the electropositive Cu atom. Subsequently, the ^*^H adsorbed on Pd atoms will protonate the ^*^CO species generated in the electroreduction of CO₂, thereby promoting the formation of CH₄. Overall, Bader charge analysis confirmed the existence of the reduction process involving Pd‐adsorbed ^*^H species. This, in turn, enhanced the exclusive selectivity toward CH₄ during the CO₂ electroreduction reaction.

To further confirm the role of Pd‐adsorbed ^*^H species, we calculated the energy contribution of Pd‐adsorbed ^*^H species to the protonation of ^*^CO (Figure 5f). With ^*^H species adsorbed on the Pd atom, the reaction‐free energy barrier for the formation of ^*^CHO decreases tremendously from 0.82 to 0.70 eV by 0.12 eV compared to that of ^*^H species adsorbed on the Cu atom, indicating the key role of Pd‐adsorbed ^*^H species during the CO_2_RR reaction. Furthermore, Cu_3_Pd has a more negative ∆G(H) (−0.275 eV) than pure Cu (−0.125 eV) (Figure S27, Supporting Information), which indicates a very high ^*^H adsorption strength.^[^ ^40^ ^]^ Significantly, the formed ^*^H species would be involved in the electroreduction process of CO_2_ to facilitate ^*^CO protonation, which was in line with the previous conclusion that we proposed.

It is well known that ^*^CO serves as a crucial intermediate in the electroreduction of CO_2_ to deep‐reduction products. The hydrogenation of ^*^CO to form ^*^CHO species represents a pathway for the generation of CH_4_. In contrast, the coupling of adjacent ^*^CO and ^*^CHO species leads to the formation of C_2_ products. On the basis of these facts, it is clear that a competitive relationship exists between the C_1_ and C_2_ reaction pathways in the CO_2_ electroreduction reaction. The Gibbs free energy changes of the CO_2_‐to‐CH_4_/C_2_H_4_ reaction on Cu and Cu_3_Pd were calculated and shown in Figure 5g and Figure S28 (Supporting Information). As depicted, the ^*^CO hydrogenation to form ^*^CHO is the rate‐determining step (RDS) for CH_4_ formation, while the RDS for the formation of C₂H₄ involves the coupling of adsorbed ^*^CO and ^*^CHO species to form ^*^COCHO. For the Cu₃Pd catalyst, the Gibbs free energy changes corresponding to the RDS for the production of CH₄ and C_2_H₄ were computed to be 0.87 and 1.16 eV (Figure 5g), respectively. This indicates that the electroreduction of CO_2_ to CH₄ is preferentially favored on Cu₃Pd. As for pure Cu, the Gibbs free energy changes associated with the RDS energy barriers for C₂H₄ and CH₄ are 0.65 and 0.96 eV (Figure S28, Supporting Information), respectively, which implies that C₂H₄ formation is more thermodynamically favored on the Cu surface than CH_4_. Besides, the activation energies for ^*^CO‐to‐^*^CHO on the Cu_3_Pd model were calculated at 1.14 eV (Figure 5h), which was lower than that calculated on Cu (1.19 eV, Figure S29, Supporting Information), suggesting the relatively faster kinetics. On the contrary, the activation energies for ^*^CO‐to‐^*^COCHO on the Cu model (0.78 eV, Figure S29, Supporting Information) were much lower than that calculated on Cu_3_Pd (1.39 eV, Figure 5h). Overall, the enhanced CH_4_ selectivity on the Cu_3_Pd catalyst was attributed to the blocking effect induced by Pd atoms and the ^*^CO protonation effect of palladium‐adsorbed ^*^H species.

## 3 Conclusion

In summary, we have demonstrated the role of Pd atoms in Cu–Pd alloy catalysts for CO_2_RR reactions. Studies on Cu and different Cu–Pd alloys have shown that the presence of adjacent Cu atoms is beneficial for the formation of C_2_ products, especially for C_2_H_4_ generation, because the active Cu sites could facilitate the C─C coupling of adjacent adsorbed ^*^CO and ^*^CHO intermediates. In contrast, the presence of Pd has a blocking effect in the electroreduction reaction of CO_2_ by separating neighboring Cu atoms, which inhibits the coupling between adjacent intermediates and thus improves the selectivity of the C_1_ pathway. Moreover, Pd atoms could serve as active centers for ^*^H adsorption, facilitating the hydrogenation process of ^*^CO intermediates to form ^*^CHO. DFT calculations also demonstrated the introduction of Pd could change the electronic structure of the catalyst, reducing the energy barrier of the reaction, and promoting the formation and transformation of ^*^CHO intermediates. It has more advantages than the Cu catalyst in electroreducing CO_2_ to CH_4_. These findings suggested an effective approach to engineer catalyst surfaces for high reactivity and high selectivity to desired products.

## 4 Experimental Section

### Synthesis of Cu Aerogels

Porous Cu aerogels were synthesized with a minor modification based on previous literature.^[^ ^41^ ^]^ In brief, 5.0 mL CuCl₂ (0.1 m) and 5.0 mL of NaOH (0.2 m) were blended into 150 mL pure DI water in a 250 mL three‐necked flask. Thereafter, the mixture was stirred vigorously for 20 min under a nitrogen atmosphere. Subsequently, 150 µL ethanol and 50 mg freshly prepared NaBH₄ (dissolved in 25 mL H₂O) were rapidly added to the solution while being strongly stirred for another 20 min. After being centrifuged and washed several times successively with ethanol and water, the porous Cu aerogel products were obtained and dried in a vacuum at 60 °C for 2 h.

### Synthesis of Cu–Pd Alloy Catalyst

For Cu_3_Pd alloy preparation, 3.75 mL of CuCl₂ (0.1 m) and 1.25 mL of Na₂PdCl₄ (0.1 m) were mixed into 150 mL of deionized water in a 250 mL three‐necked flask. Then, 5.0 mL of NaOH (0.2 m) was added to the above solution, and the mixture was stirred for 20 min under a nitrogen atmosphere. After that, 50 mg of fresh NaBH₄ (dissolved in 25 mL of H₂O) and 150 µL of ethanol were quickly added to the solution under uniform stirring for 20 min. Subsequently, after centrifuging and washing three times successively with ethanol and water, the Cu₃Pd aerogel products were obtained and dried in a vacuum at 60 °C for 2 h. The CuPd alloys were synthesized using a similar procedure, except that the proportions of the CuCl₂ solution and the Na₂PdCl₄ solution were adjusted, with 2.5 mL of CuCl₂ and 2.5 mL of Na₂PdCl₄ used for it. Finally, the Cu₃Pd aerogels were placed in a tube furnace and calcined at 500 °C in a reducing atmosphere to obtain the Cu₃Pd alloy.

## Conflict of Interest

The authors declare no conflict of interest.

## Supporting information



Supporting Information

## Data Availability

Research data are not shared.
