# Supplementary material for: Highly Selective Electroreduction of CO2 to CH4 on Cu–Pd Alloy Catalyst: the Role of Palladium‐Adsorbed Hydrogen Species and Blocking Effect
Source: Adv Sci (Weinh). 2025 Mar 26;12(19):2417247. doi: 10.1002/advs.202417247 (PMC12097132; doi:10.1002/advs.202417247)
Supplement: Supplementary file 1 — Supporting Information [file ADVS-12-2417247-s001.docx]

*Supplementary Information*

**Highly Selective Electroreduction of CO_2_ to CH_4_ on Cu-Pd Alloy Catalyst: The Role of Palladium-adsorbed Hydrogen Species and Blocking Effect**

*Jinyan Huang, Ye Yang, Xuexue Liang, Bing Chen, Yue Shen, Yan Chen, Jielian Yang, Yinglin Yu, Fang Huang, Huibing He, Peican Chen*, Liya Zhou*, Anxiang Guan**

School of Chemistry and Chemical Engineering, Guangxi Key Laboratory of Electrochemical Energy Materials, State Key Laboratory of Featured Metal Materials and Life-cycle Safety for Composite Structures, Guangxi Key Laboratory of Petrochemical Resource Processing and Process Intensification Technology, Guangxi Colleges and Universities Key Laboratory of Applied Chemistry Technology and Resource Development, Guangxi University, Nanning 530004, China.

*Corresponding authors.

Email address: peicanchen@gxu.edu.cn, zhouliya@gxu.edu.cn, [Axguan24@gxu.edu.cn](mailto:Axguan24@gxu.edu.cn)

**Experimental Section**

**Materials.** Cupric chloride (CuCl_2_), sodium tetrachloropalladate (Na_2_PdCl_4_), sodium borohydride (NaBH_4_), sodium hydroxide (NaOH), ethanol, deionized water, potassium hydroxide (KOH), Nafion (5 wt%). All materials were used directly as they were obtained without undergoing any further purification. Deionized water (from Millipore, with a resistivity of 18.2 MΩ) was used in all the experiments.

**Electrochemical measurements.** Electroreduction measurements were conducted in a three-electrode flow cell at ambient conditions. A gas-diffusion electrode painted with IrO_2_ catalyst (1 mg cm^-2^) used as the counter electrode and another gas-diffusion electrode painted with catalyst used as the work electrode. In this work, all of the voltages were converted to the reversible hydrogen electrode (RHE) scale, using the following formula: *E_RHE_* = *E_Ag/AgCl_* + 0.210 + 0.0591×pH. For the Faradaic efficiency analysis, gas and liquid products were quantified by in-line gas chromatograph (Shanghai Ramiin GC 2060) and ^1^H NMR on Bruker AVANCEAV III HD 500, respectively.

**Computational method.** Figure 5a shows the theoretical models of the studied systems. The structural models of pure Cu, Cu_3_Pd are constructed as 2×2 periodic supercells, which contains three atomic layers with the bottom layer of the slab were fixed and the upper two layers were fully relaxed. Here, we considered Cu (100) and Cu_3_Pd (111) facets in our models, which have been identified as being active in the CO₂ reduction reaction. First-principles calculations were performed by applying the projector-augmented wave (PAW) scheme ^[S1]^ within the Vienna ab initio Simulation Package (VASP). ^[S2, S3]^ The generalized gradient approximation (GGA) with Perdew–Burke–Ernzerhof (PBE) parameterization was used as the exchange-correlation functional. ^[S4]^ The kinetic energy cut-off for all calculations was set as 400 eV. Monkhorst–Pack 3 × 3 × 1 meshes were used to sample for integration over the first Brillouin zone for the 2 × 2 supercell. The structures were allowed to relax until the total energy was less than 1 × 10^−8^ eV and the Hellmann–Feynman force was less than 0.02 eV/Å. Vacuum layers with a thickness of at least 15 Å were used to avoid interactions between adjacent cells. The Gibbs free energy was calculated at 298.15 K by the VASPKIT code.

**Figures**


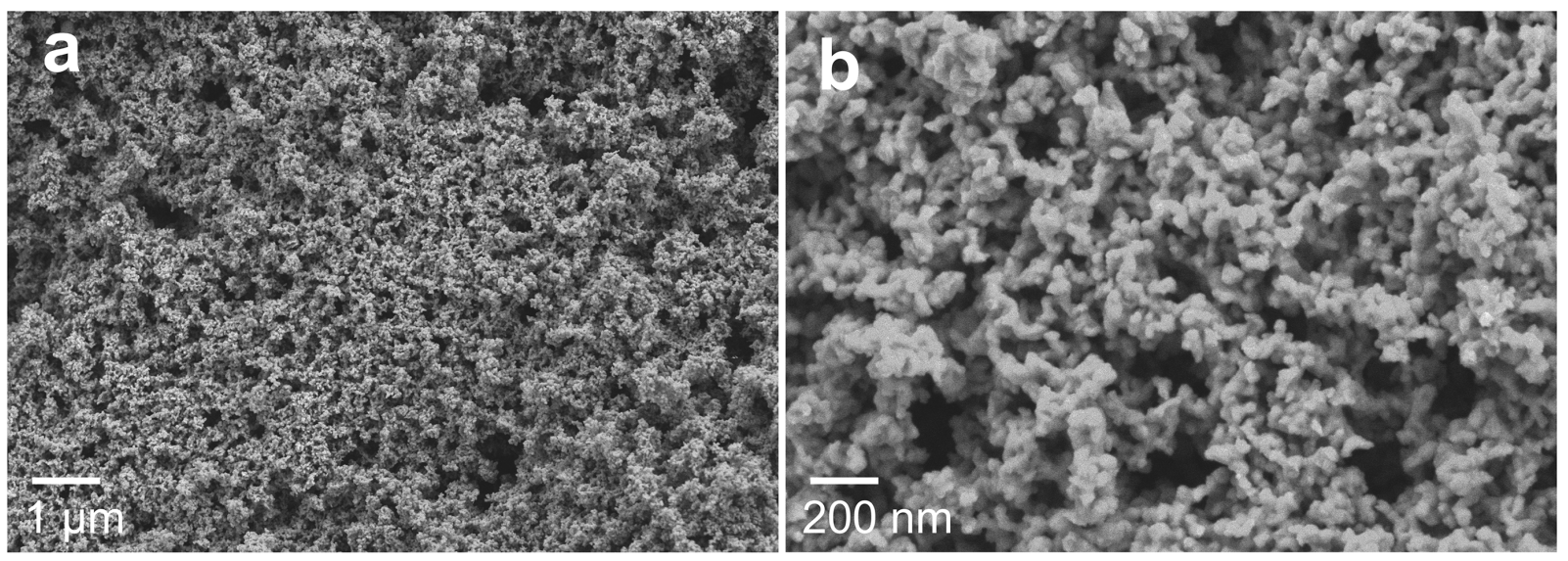


**Figure S1.** Low- (a) and high-resolution (b) SEM images of Cu.


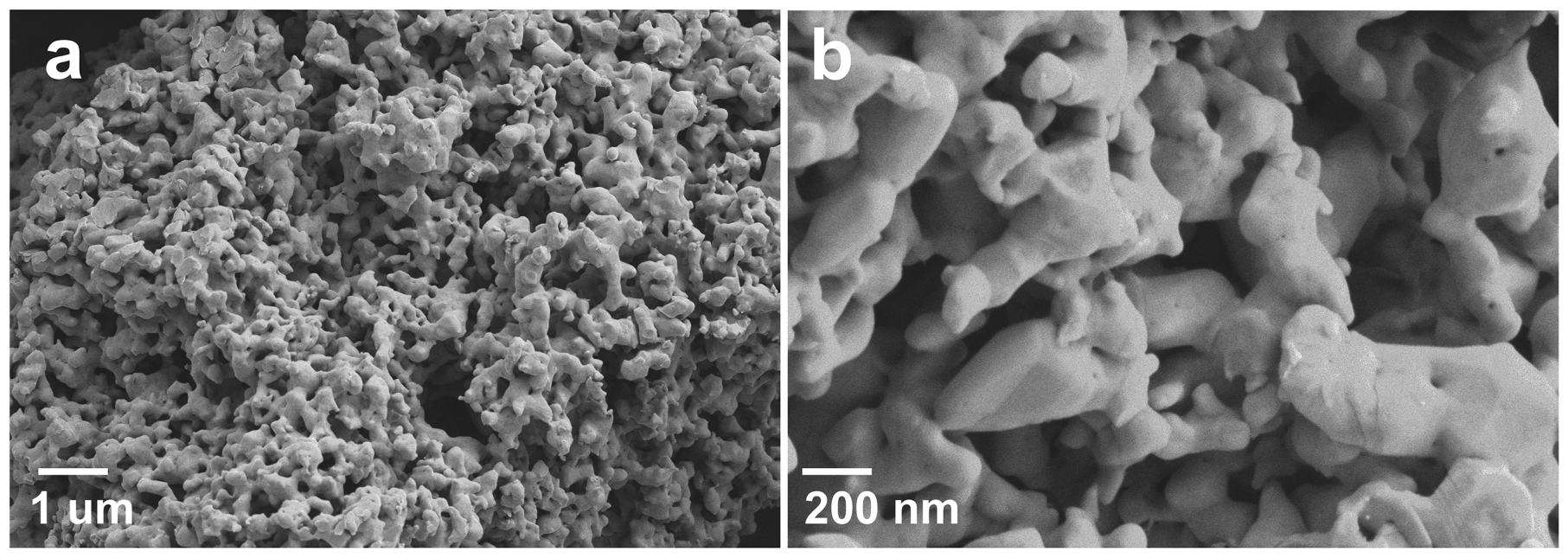


**Figure S2.** Low- (a) and high-resolution (b) SEM images of Cu_3_Pd.


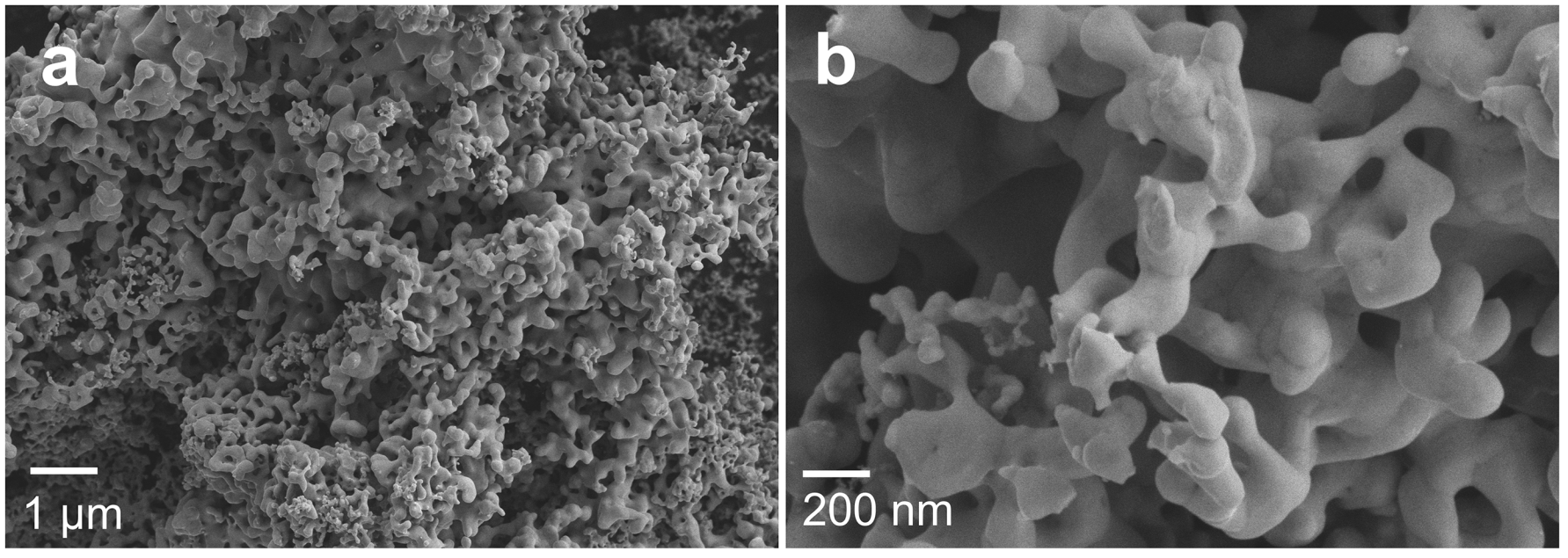


**Figure S3.** Low- (a) and high-resolution (b) SEM images of CuPd.


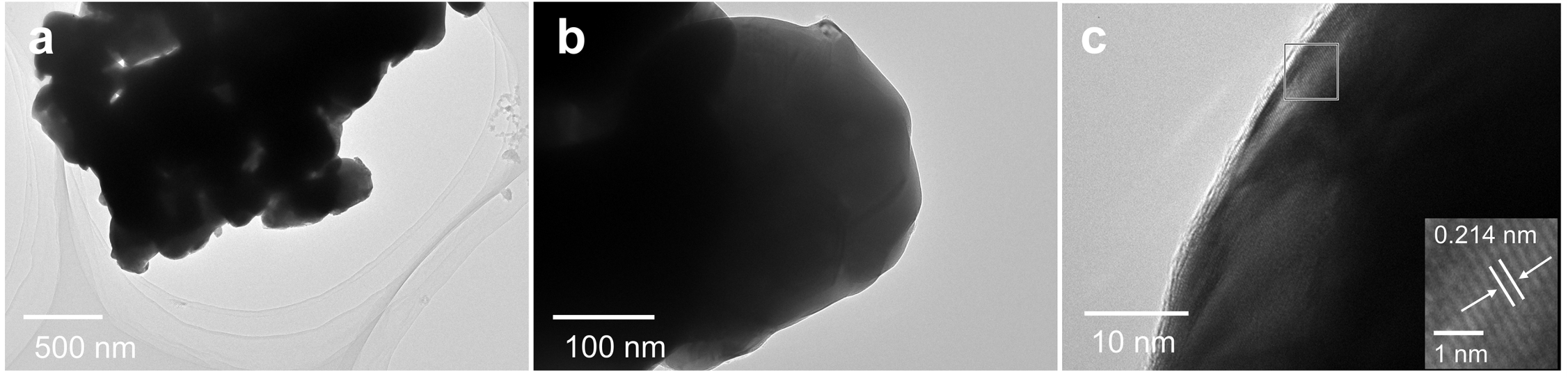


**Figure S4.** Low- (a) and high-resolution (b) TEM images of Cu_3_Pd.


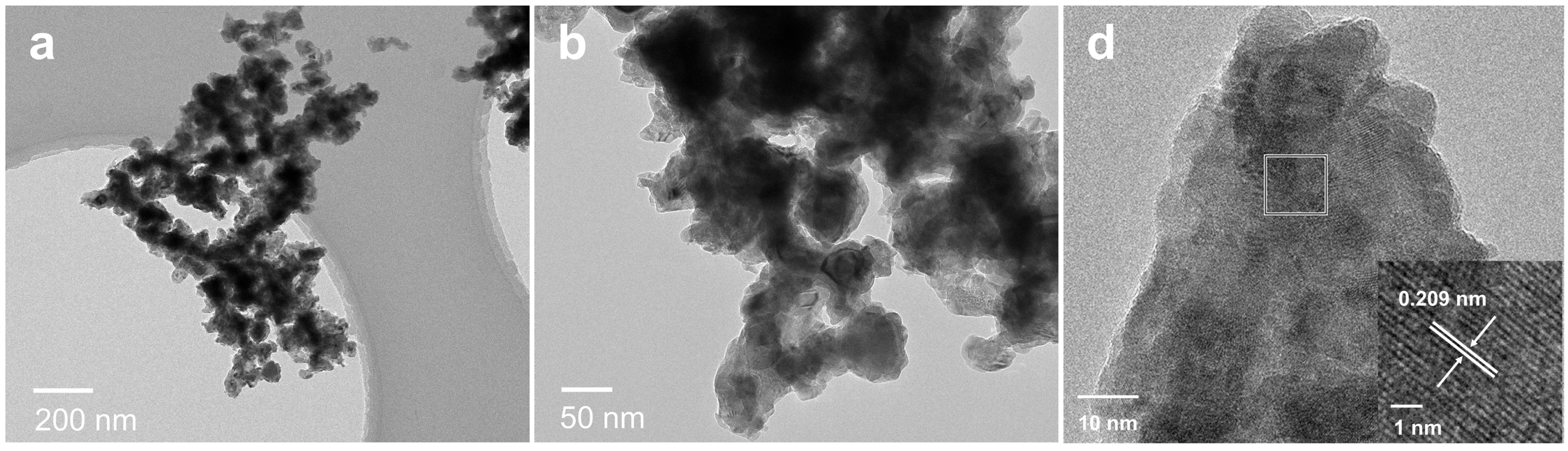


**Figure S5.** TEM images of Cu sample in different resolutions. The lattice spacing of 0.209 nm corresponds to the Cu (111) plane.


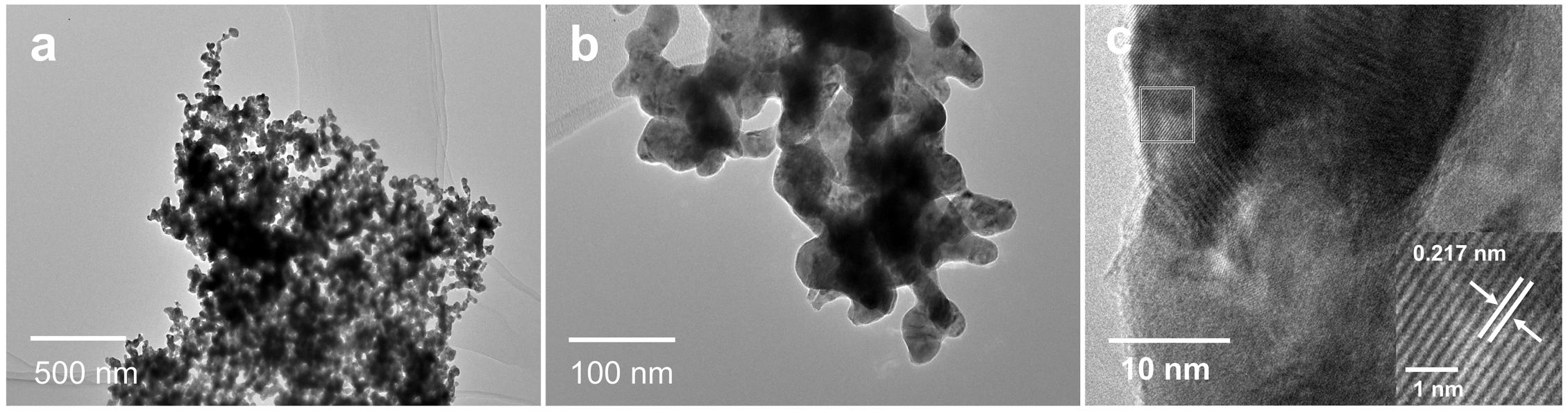


**Figure S6.** TEM images of CuPd sample in different resolutions. The lattice spacing of 0.217 nm corresponds to the CuPd (111) plane.


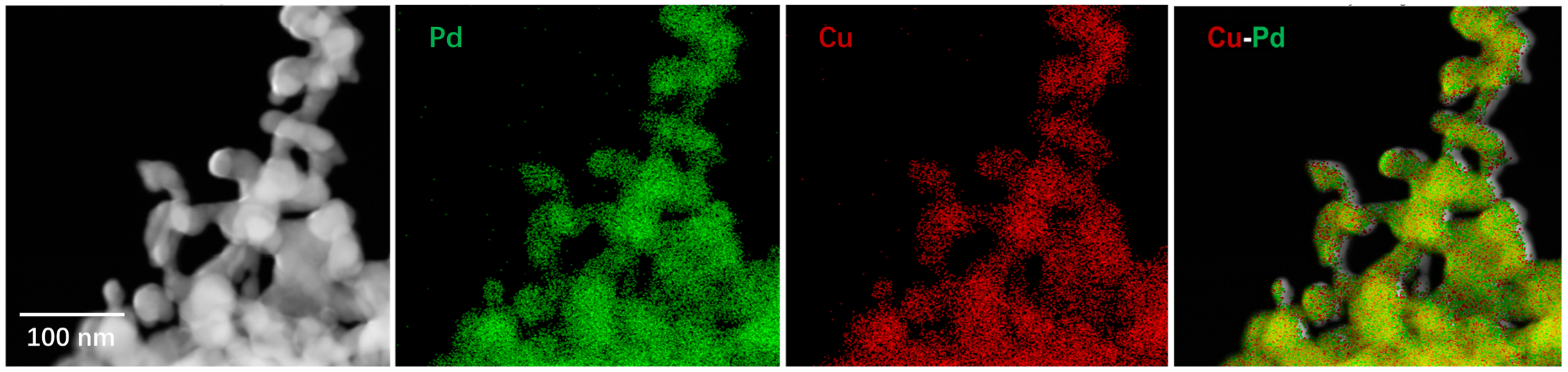


**Figure S7.** EDS elemental mappings of CuPd alloy.


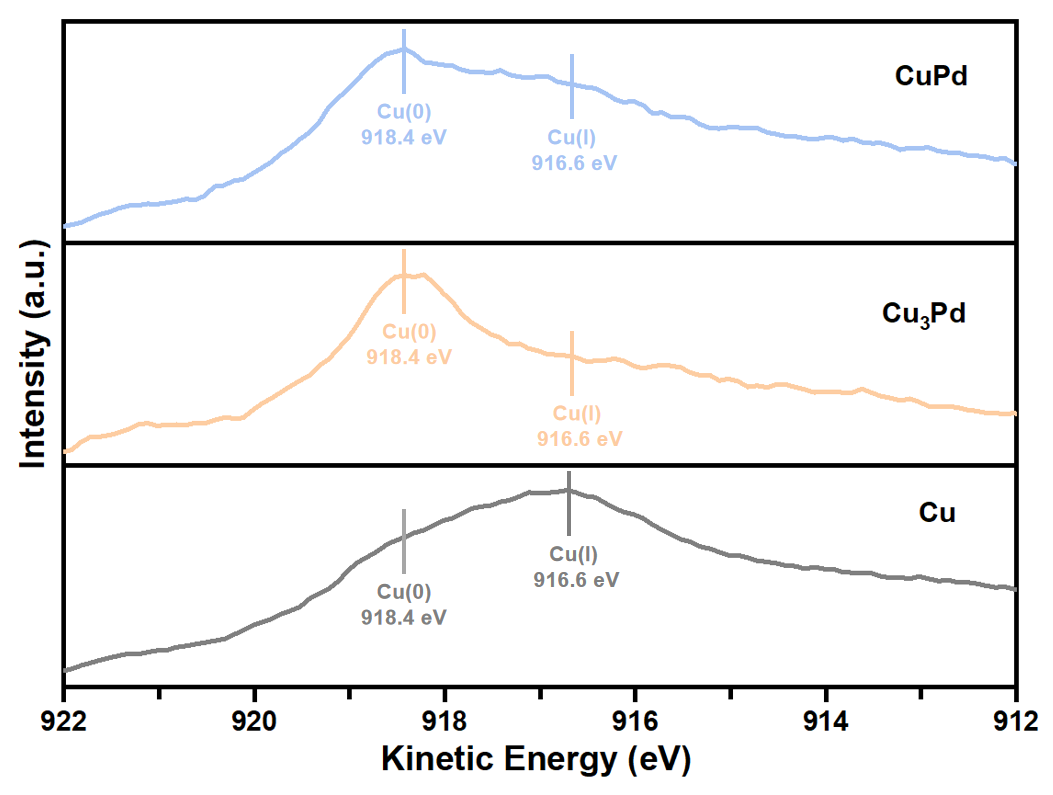


**Figure S8.** Cu LMM Auger spectra of Cu, Cu_3_Pd and CuPd samples


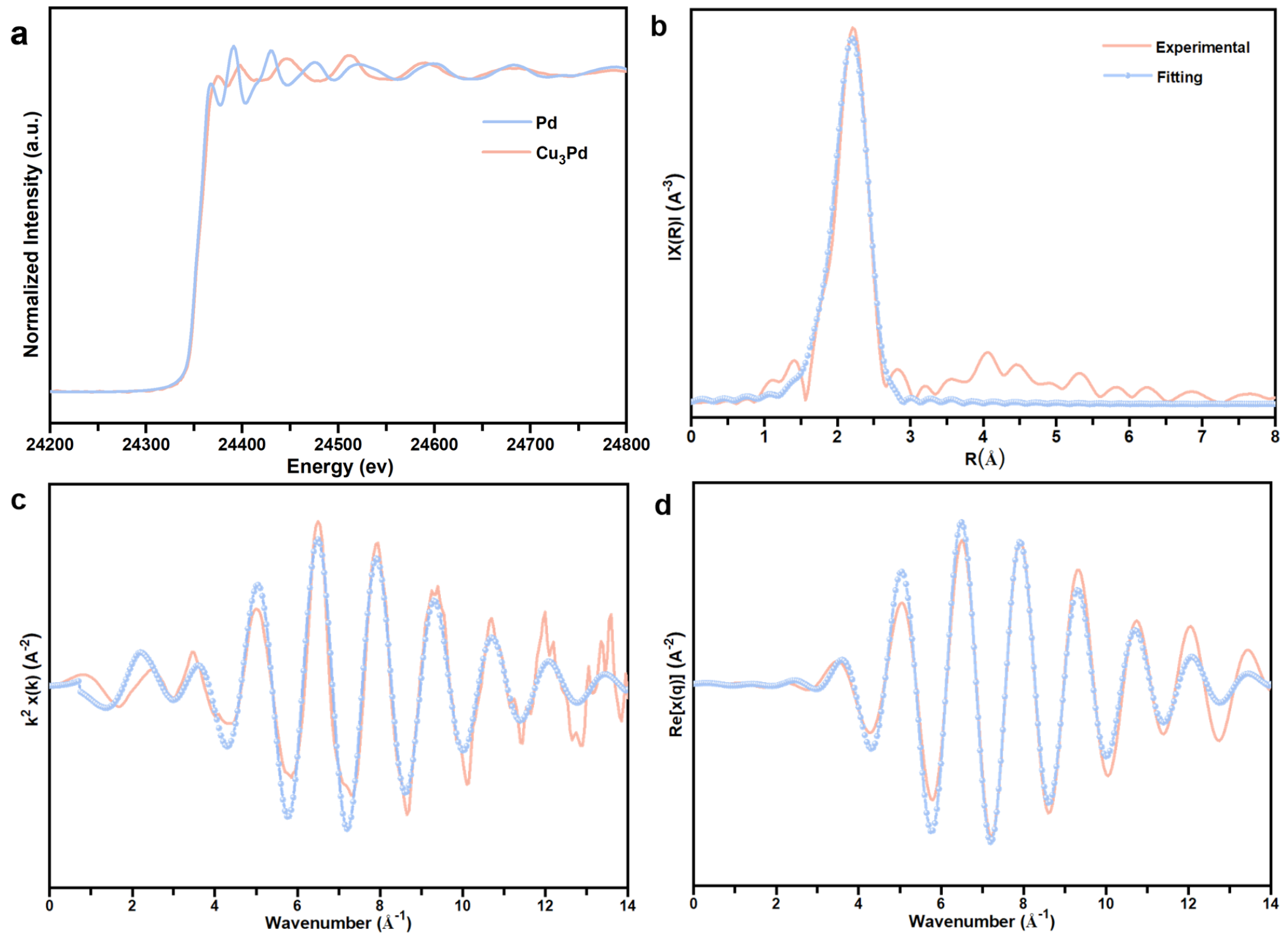


**Figure S9.** (a) Pd-edge XANES spectra of Pd foil, Cu_3_Pd. (b) EXAFS fitting curve for Cu_3_Pd at Pd K edge. (c) FT-EXAFS k space fitting curves of Cu_3_Pd alloys at Pd K-edge. (d) FT-EXAFS q space fitting curves of Cu_3_Pd alloys at Pd K-edge.


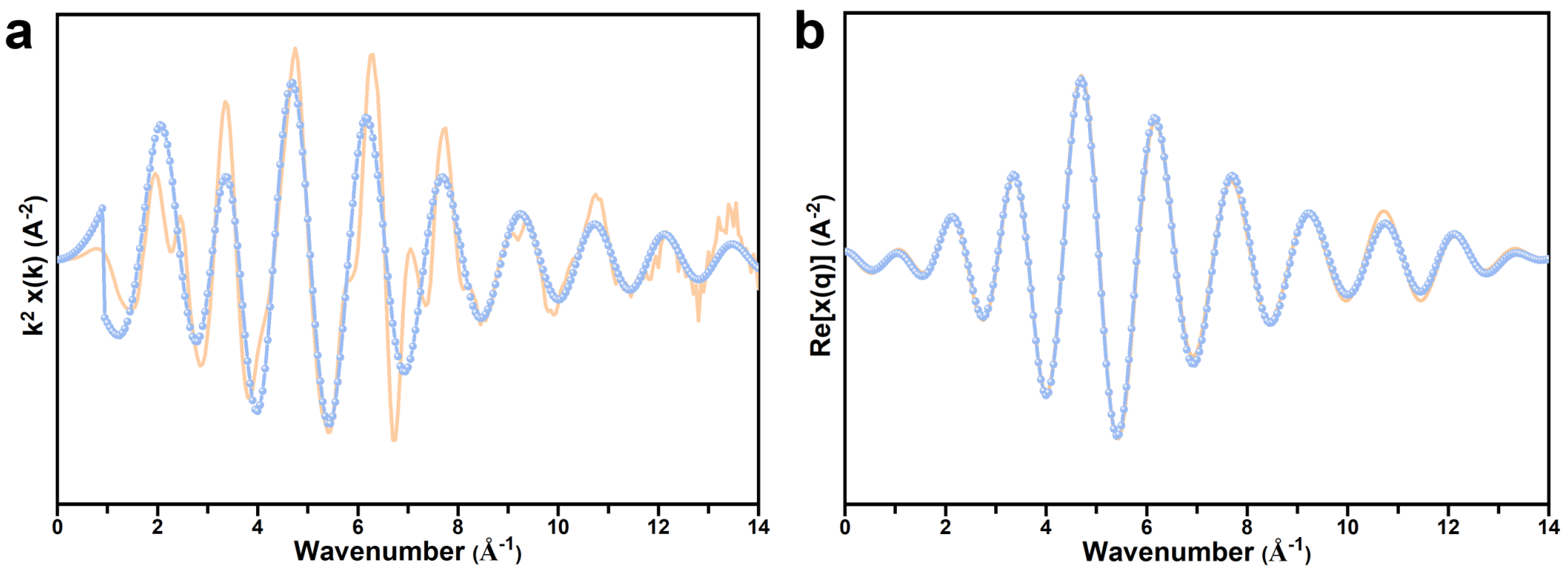


**Figure S10.** (a) Fourier-transform extended X-ray absorption fine structure (FT-EXAFS) k space fitting curves of Cu_3_Pd at Cu K-edge. (b) FT-EXAFS q space fitting curves of Cu_3_Pd alloys at Cu K-edge.


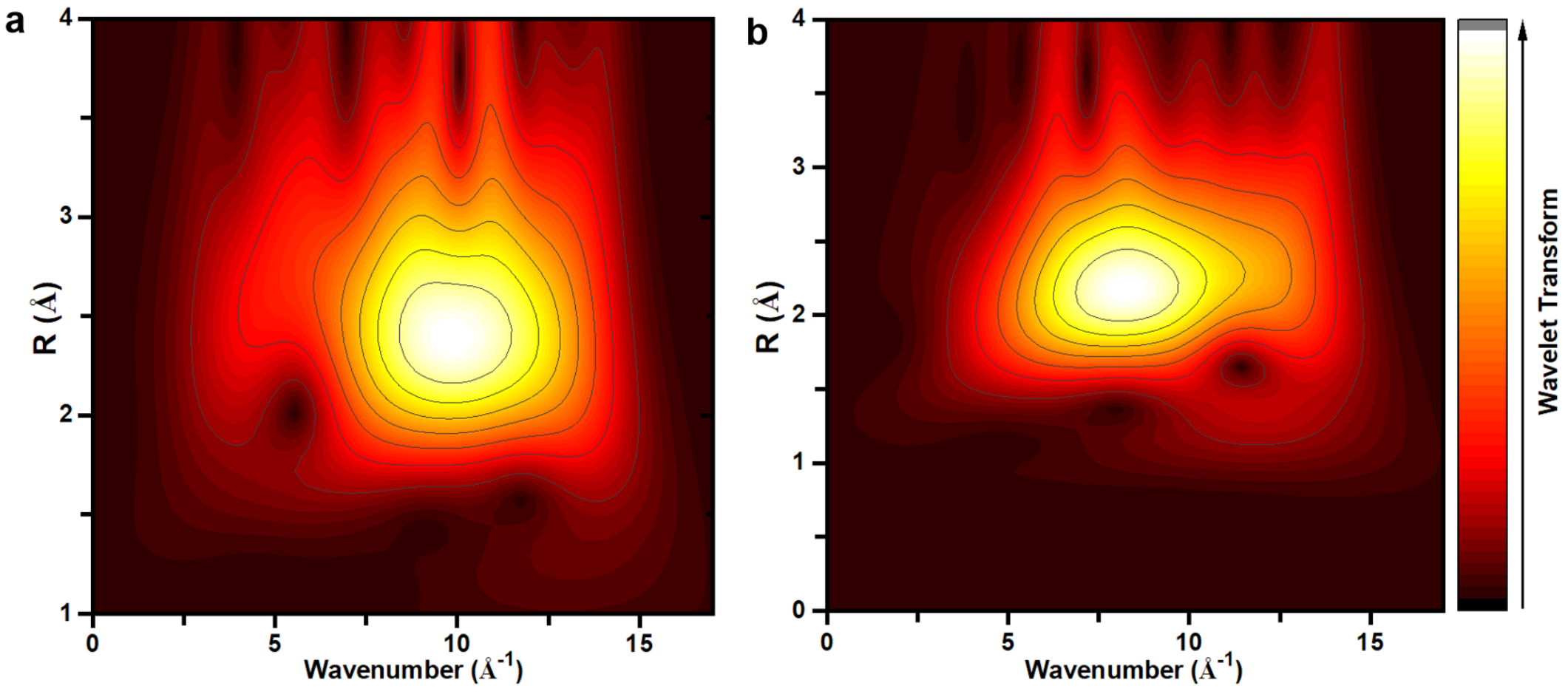


**Figure S11.** Wavelet transform images of EXAFS data at Pd K-edge with the optimized Morlet parameter for (a) Pd foil and (b) Cu_3_Pd.


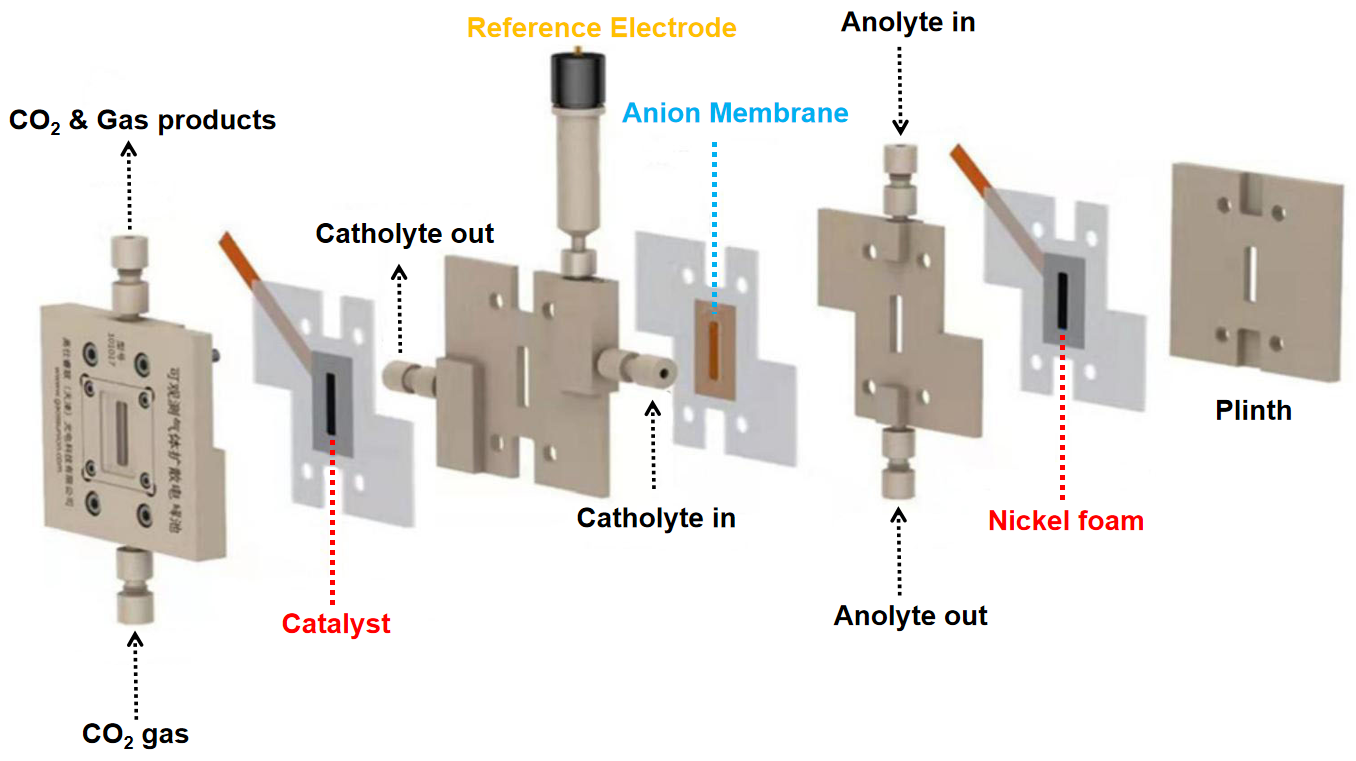


**Figure S12.** Schematic illustration of a flow cell for CO_2_ electroreduction.


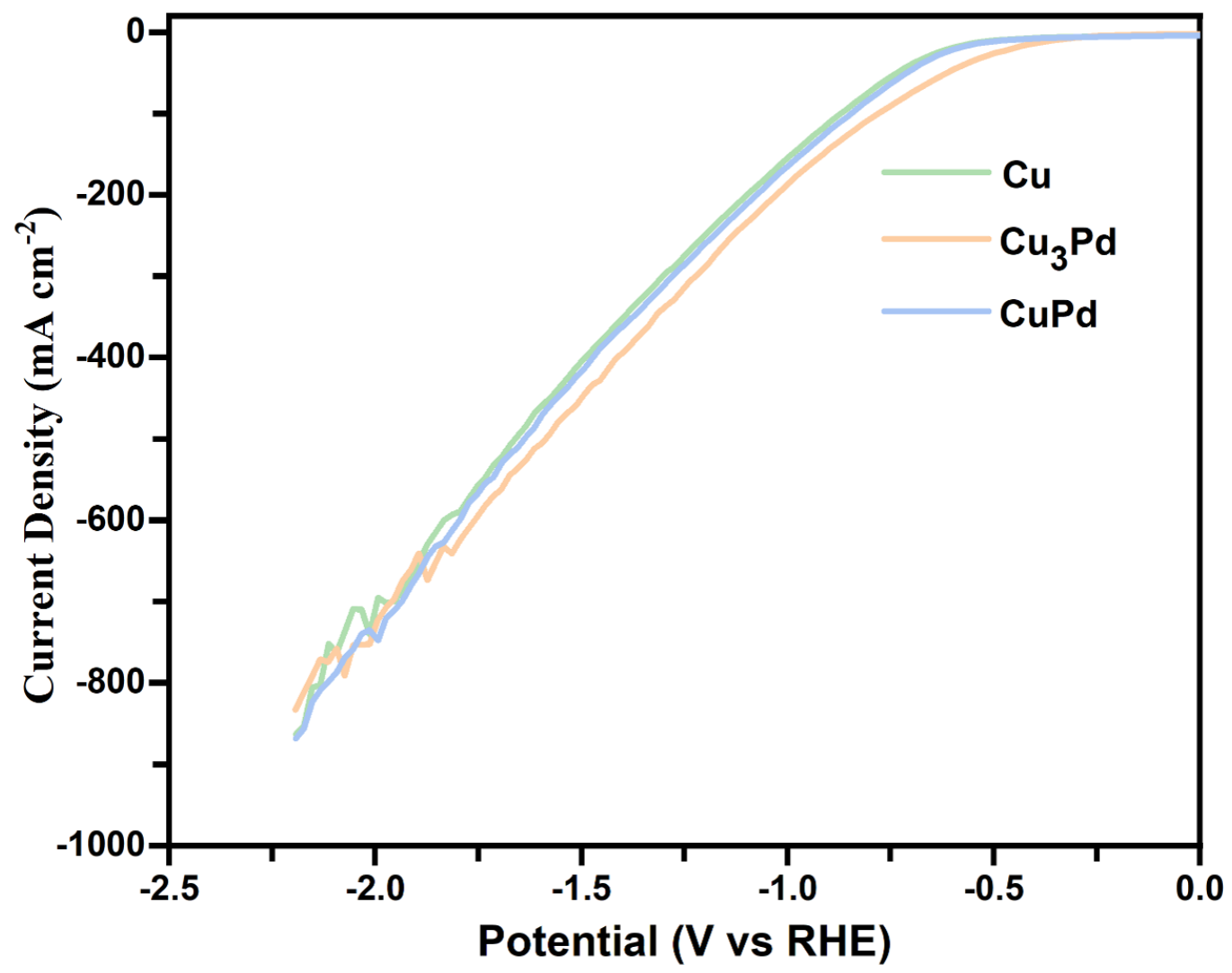


**Figure S13.** LSV curves of Cu, Cu_3_Pd and CuPd catalysts were measured in CO_2_-flowed KOH electrolyte.


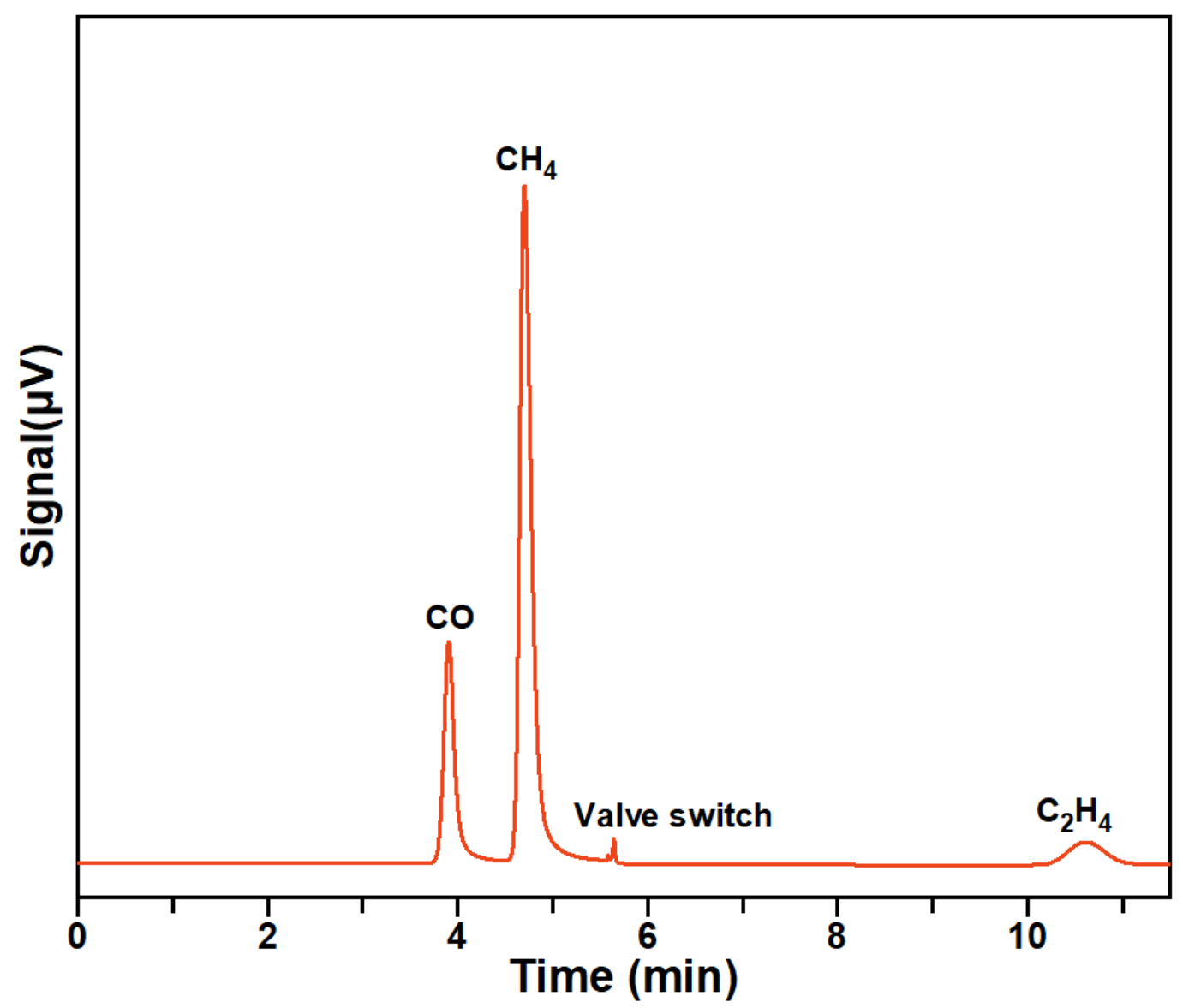


**Figure S14.** Gas-chromatography spectra of gas products for CO_2_ electroreduction on Cu_3_Pd catalyst.


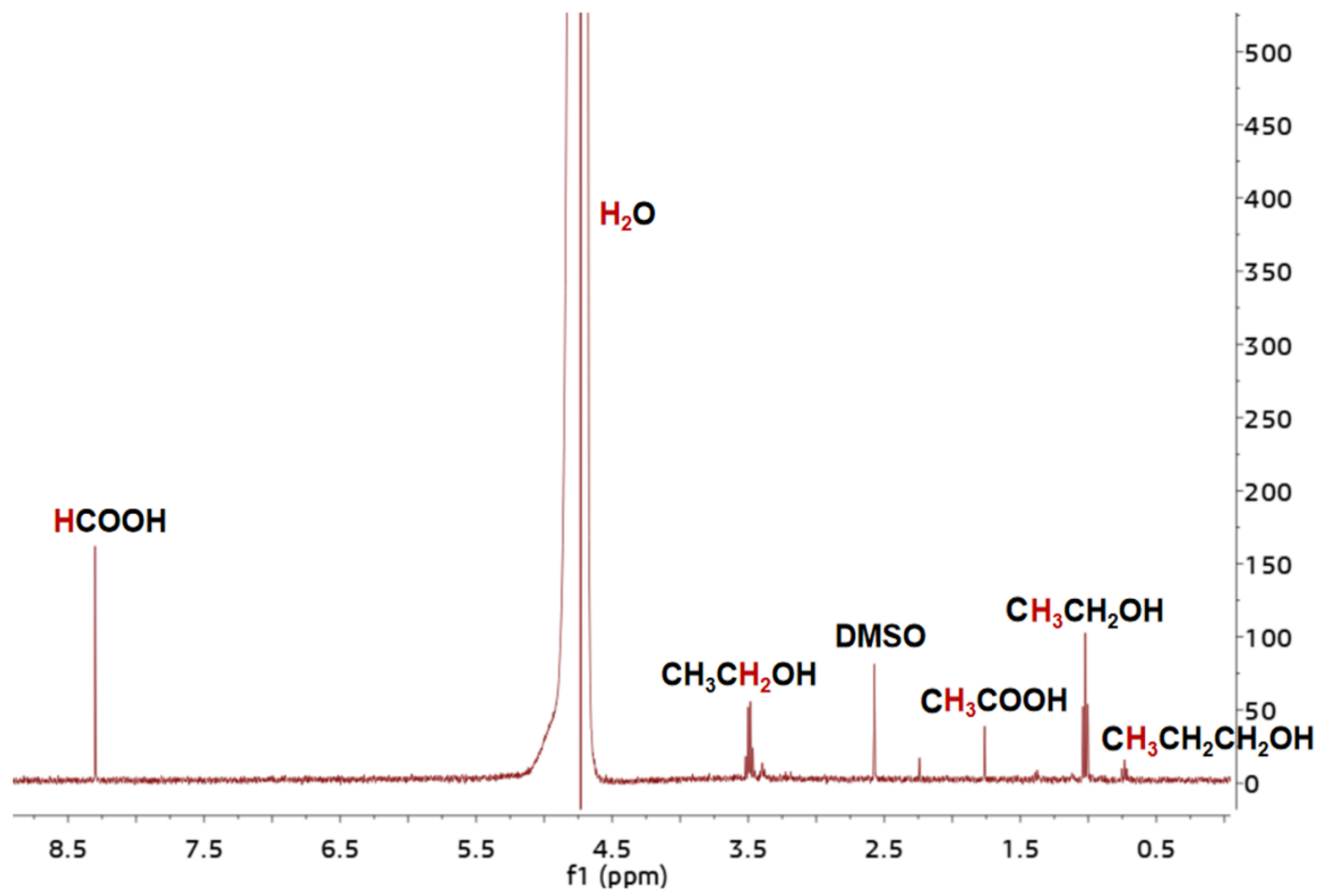


**Figure S15.** ^1^H-NMR spectrum of liquid products for CO_2_ electroreduction on Cu_3_Pd catalyst.


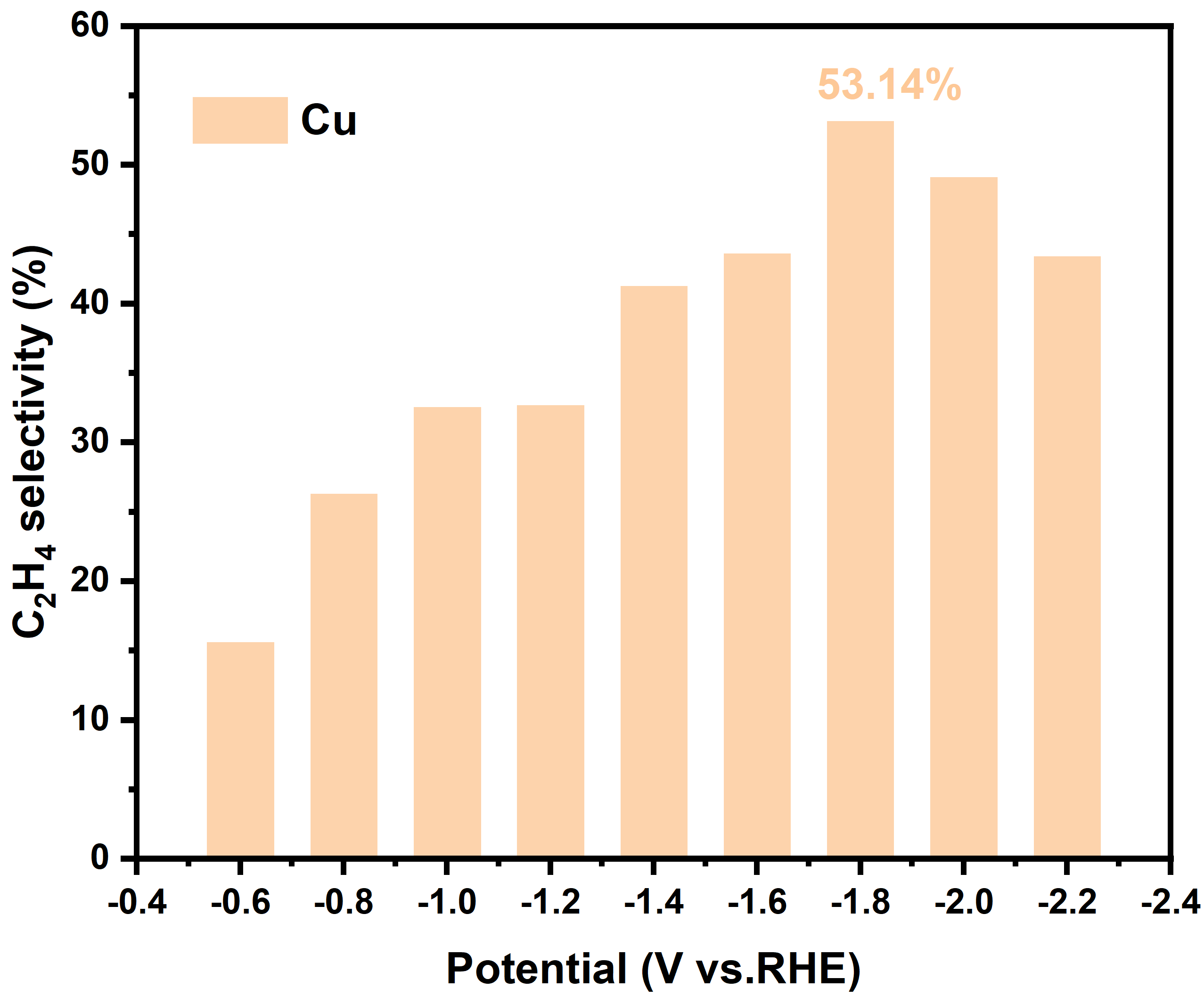


**Figure S16.** C_2_H_4_ selectivity of Cu catalyst.


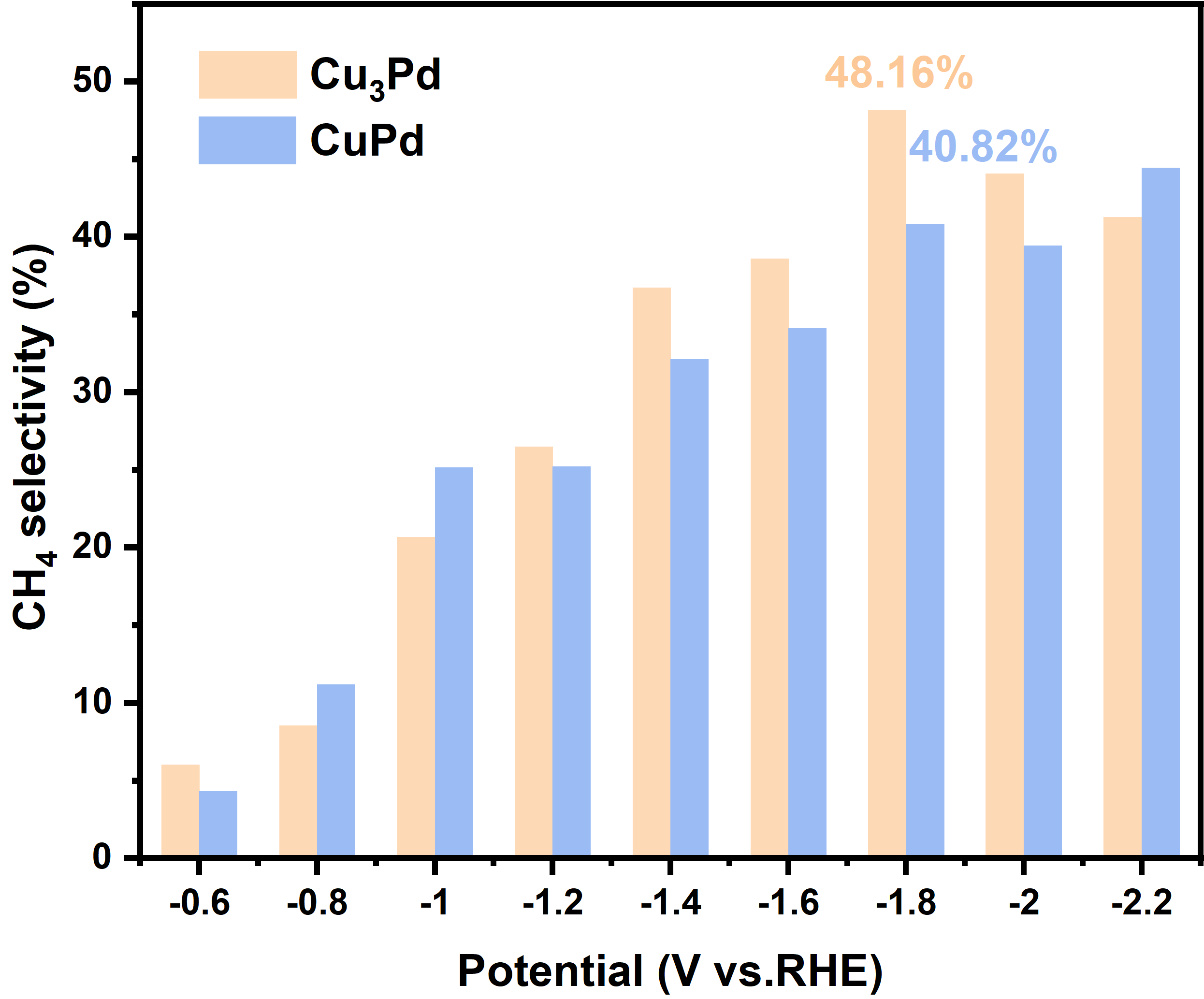


**Figure S17.** CH_4_ selectivity of Cu_3_Pd and CuPd catalysts.


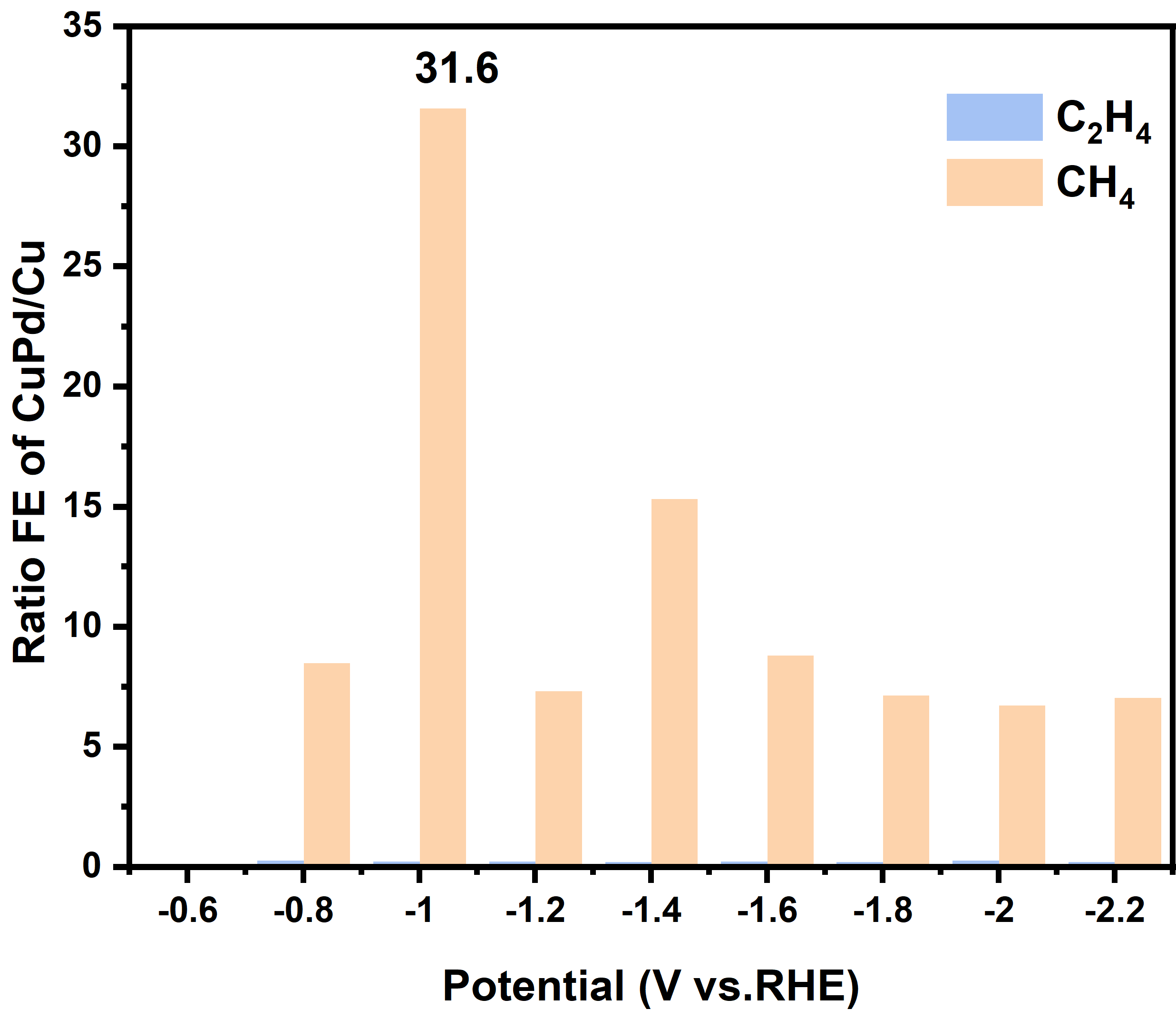


**Figure S18.** The ratios of FE_C2H4_ and FE_CH4_ between CuPd and Cu alloys.


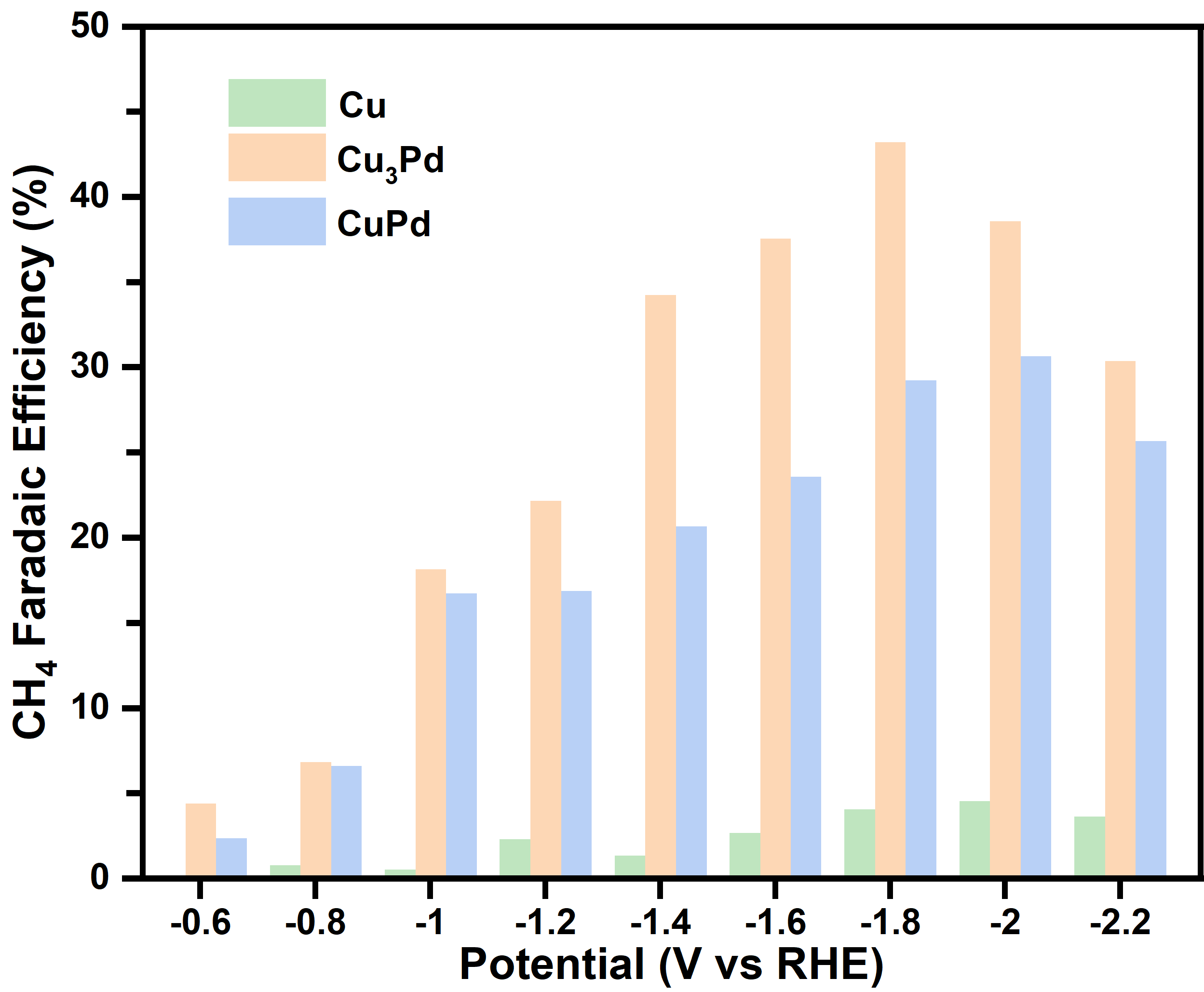


**Figure S19.** The comparison of FE_CH4_ for Cu, Cu_3_Pd and CuPd catalysts


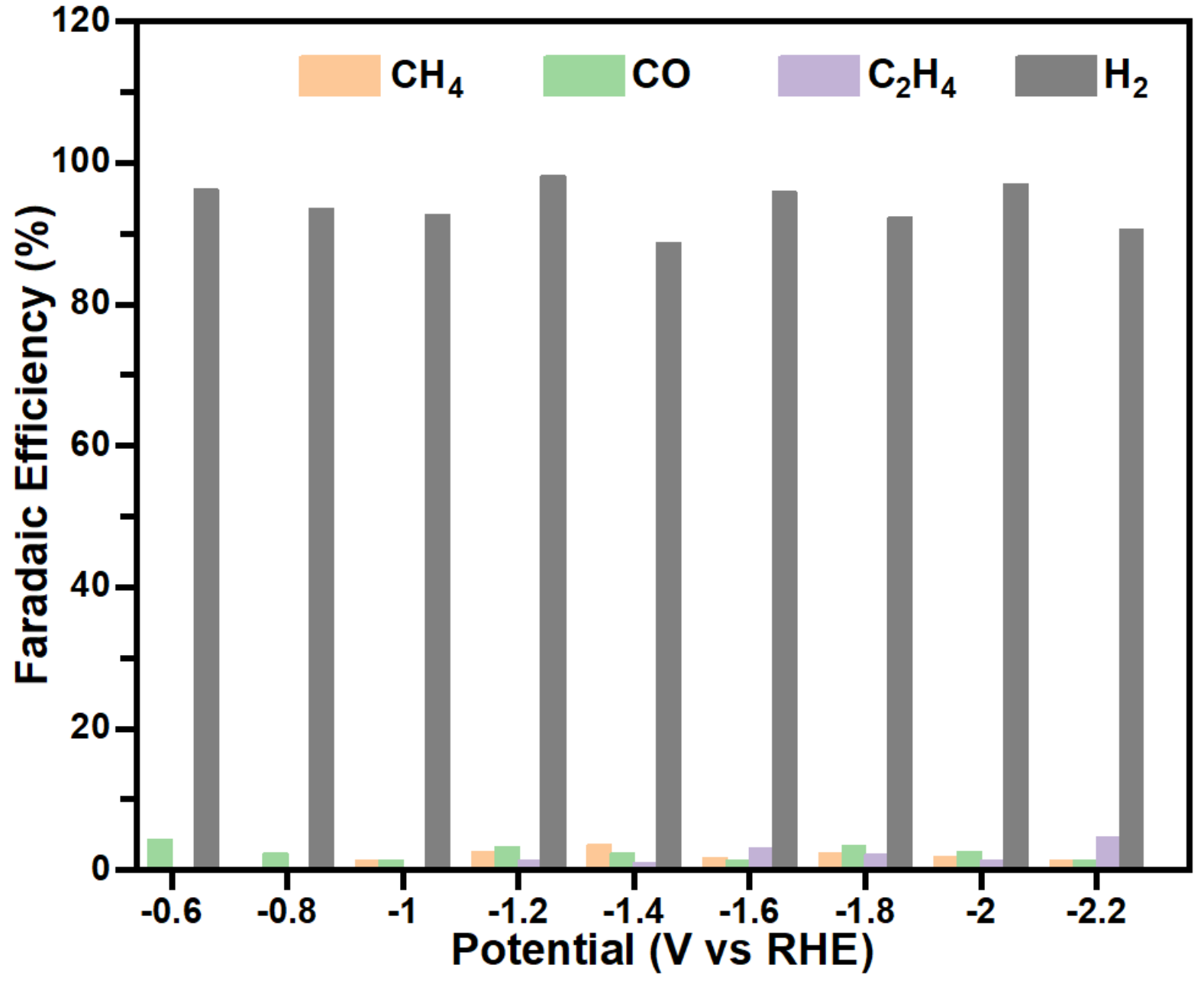


**Figure S20.** Gas products of CO_2_ electroreduction on the Pd catalyst.


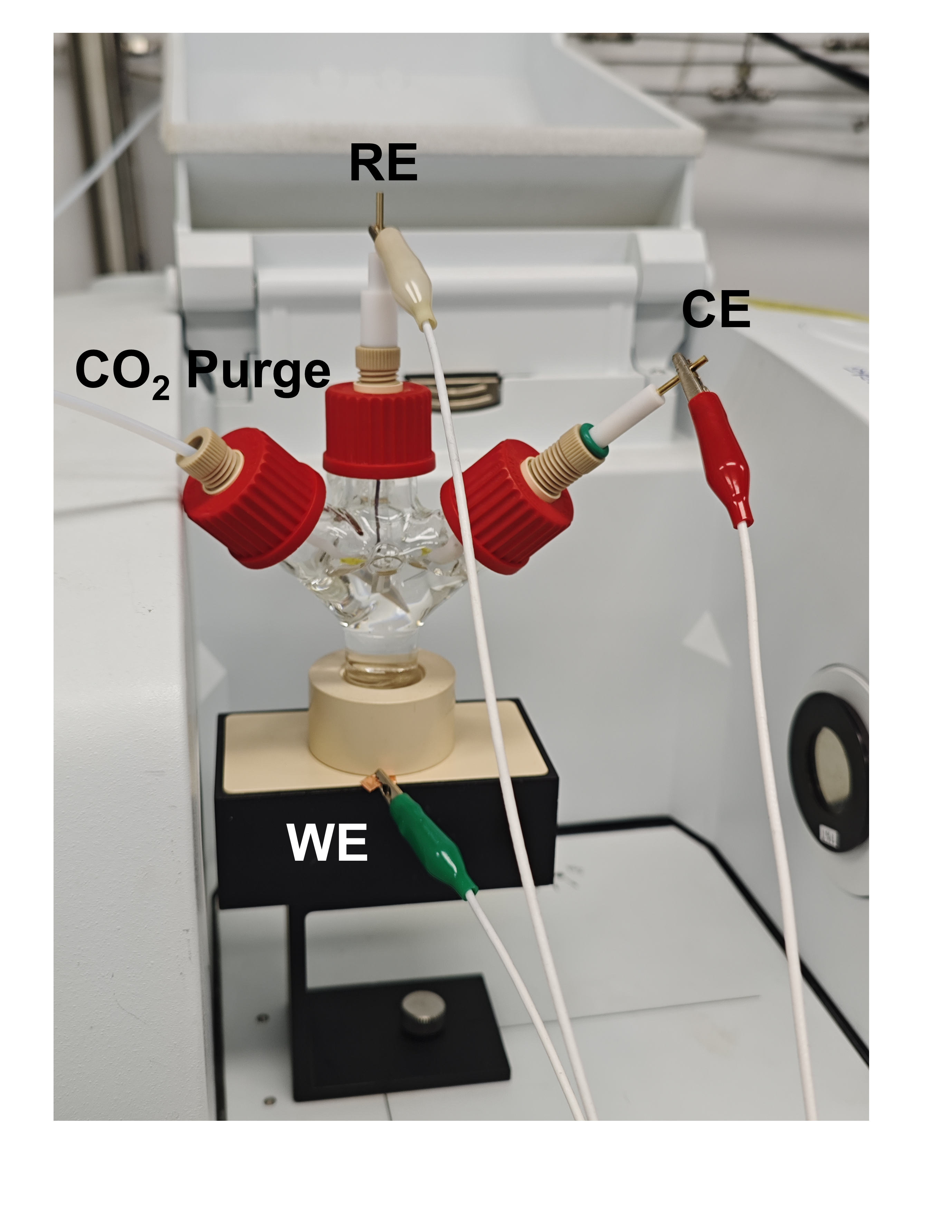


**Figure S21.** Operando ATR-SEIRS test device photograph. WE: working electrode, CE: Counter electrode, RE: Reference Electrode.

**
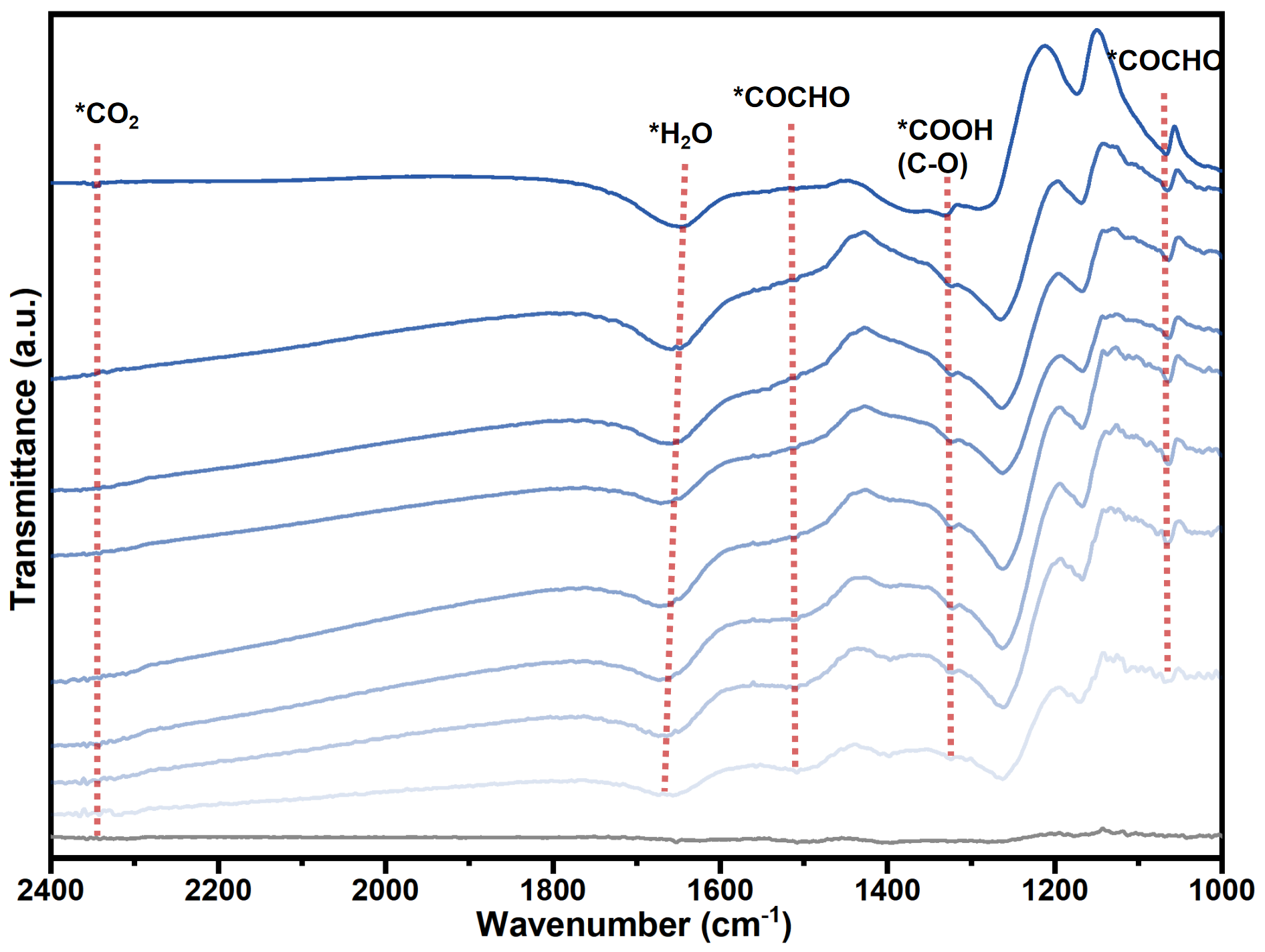
**

**Figure S22.** Operando ATR-SEIRAS spectra of Cu catalyst for CO_2_ electroreduction at different potentials in CO_2_-saturated 0.5 M KHCO_3_ electrolyte.

**
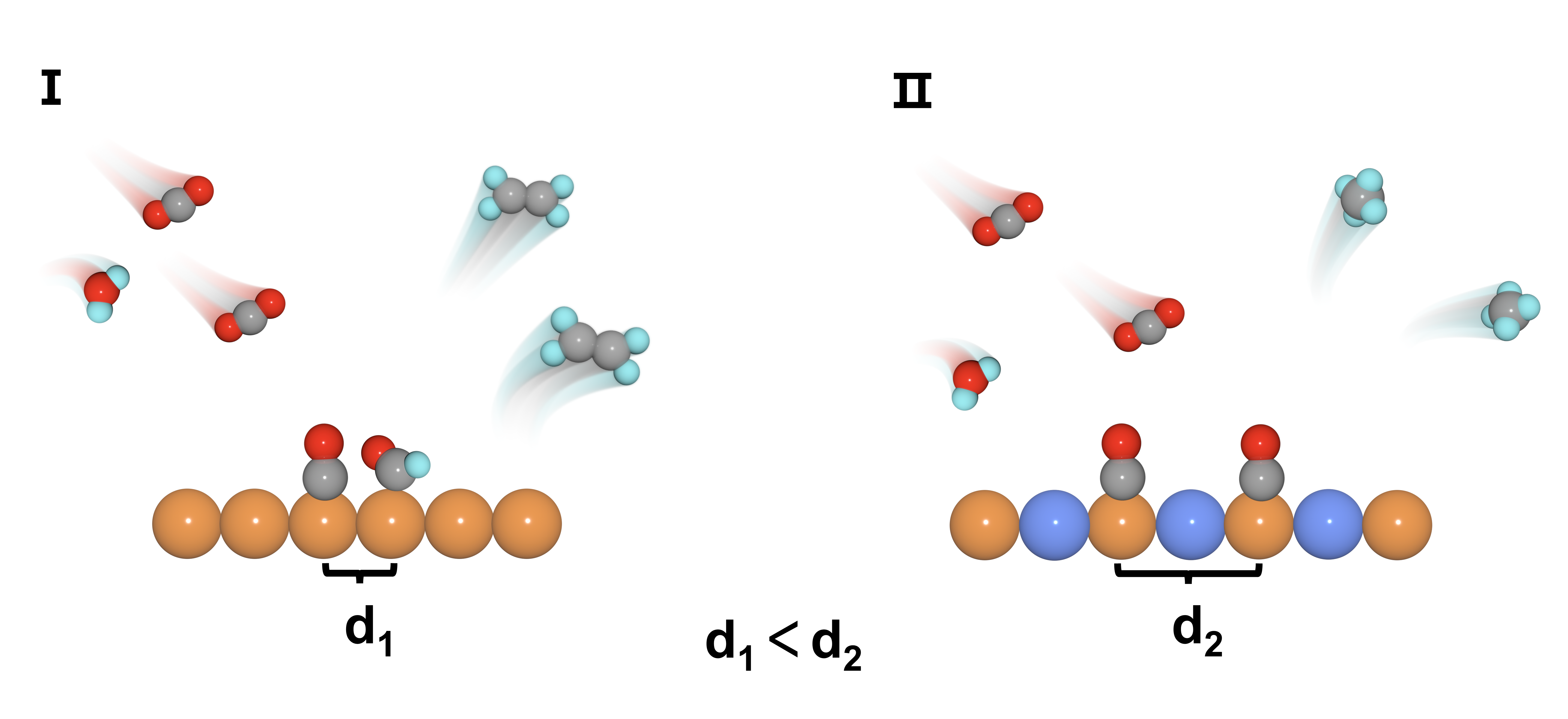
**

**Figure S23.** Schematic illustration for CH_4_ formation on Cu and Cu_3_Pd catalysts: the orange, blue and azure spheres represent Cu, Pd and H atoms, respectively.


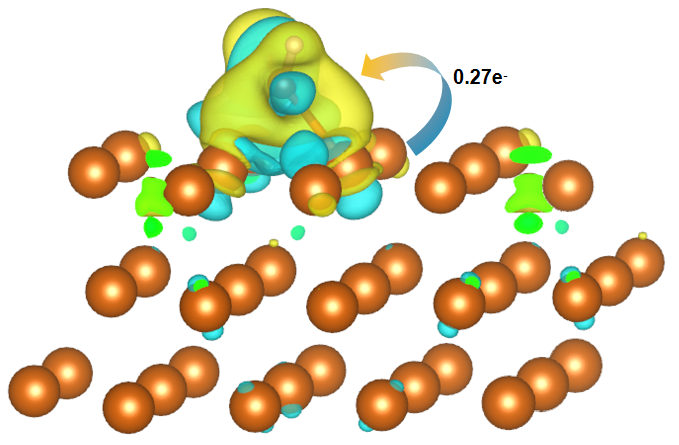


**Figure S24.** Differential charge density distributions of Cu with the *CHO intermediates. The orange, grey, red and white spheres represent Cu, C, O and H atoms, respectively.


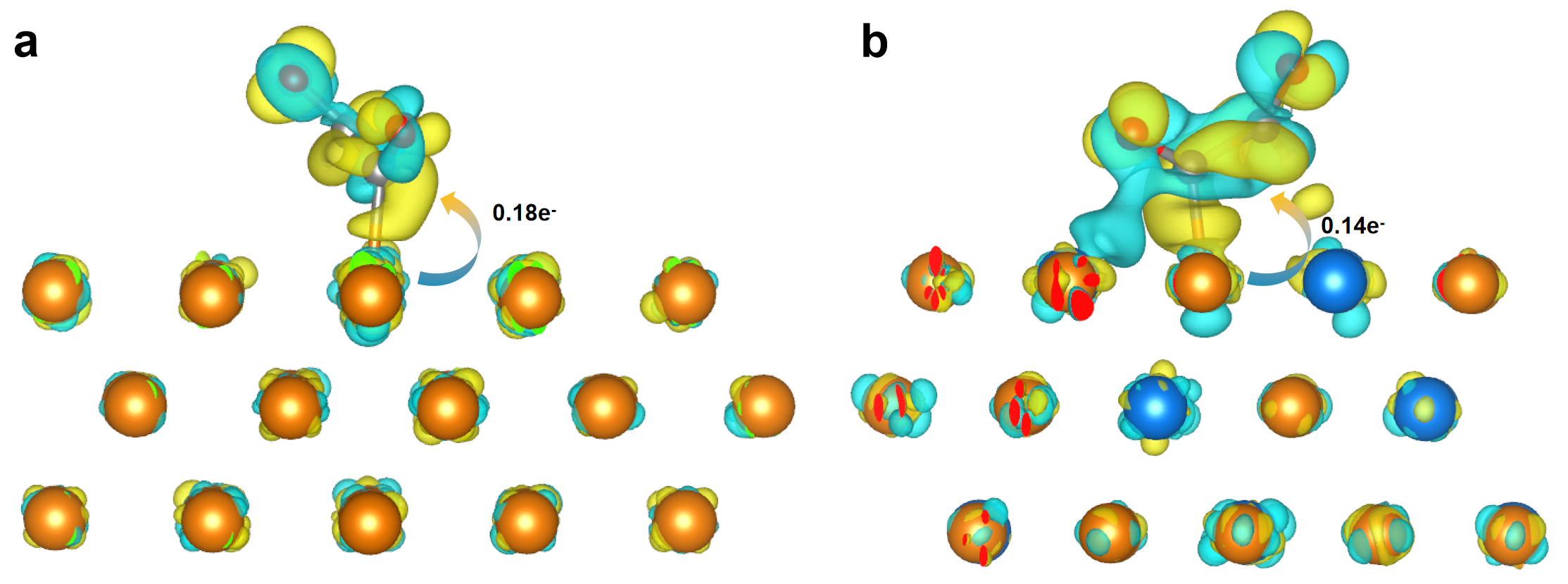


**Figure S25.** Differential charge density distributions of (a) Cu and (b) Cu_3_Pd with the *COCHO intermediates. The orange, blue, grey, red and white spheres represent Cu, Pd, C, O and H atoms, respectively.


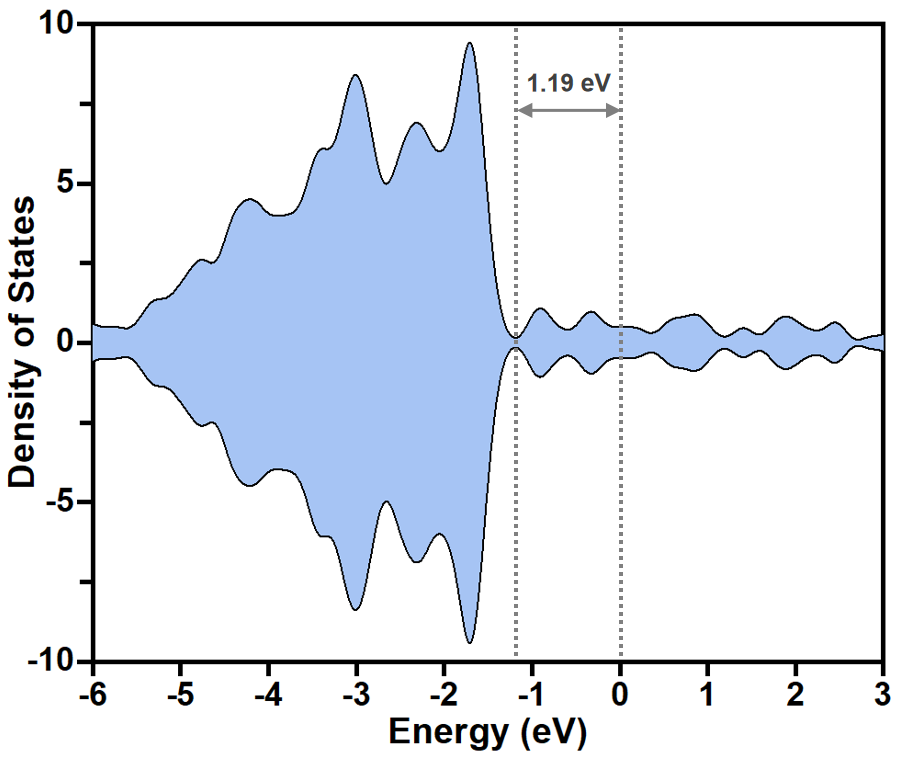


**Figure S26.** The calculated total density of states for pure Cu.


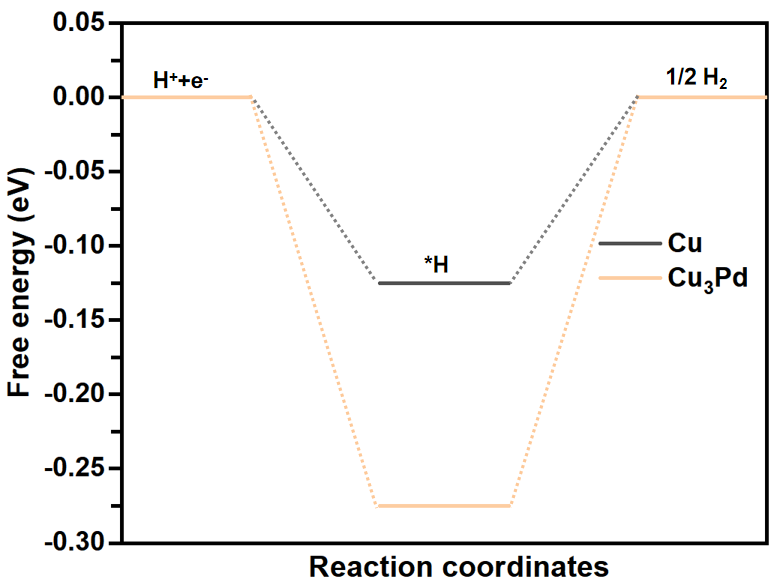


**Figure S27.** The calculated free energy diagrams of HER.


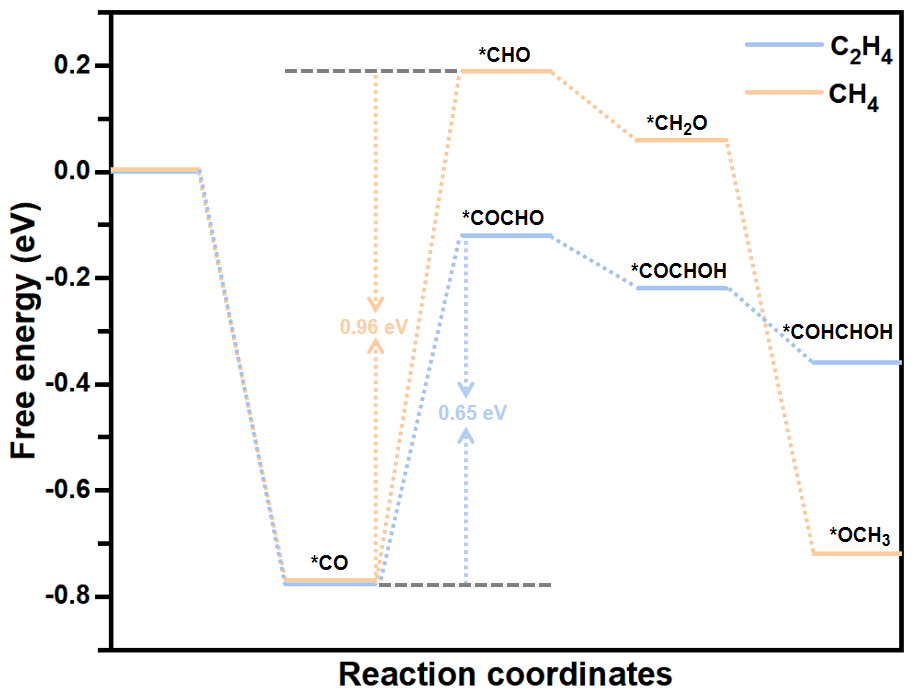


**Figure S28.** Reaction paths and free energy diagrams of CO_2_ reduction to CH_4_ and C_2_H_4_ on Cu catalysts.


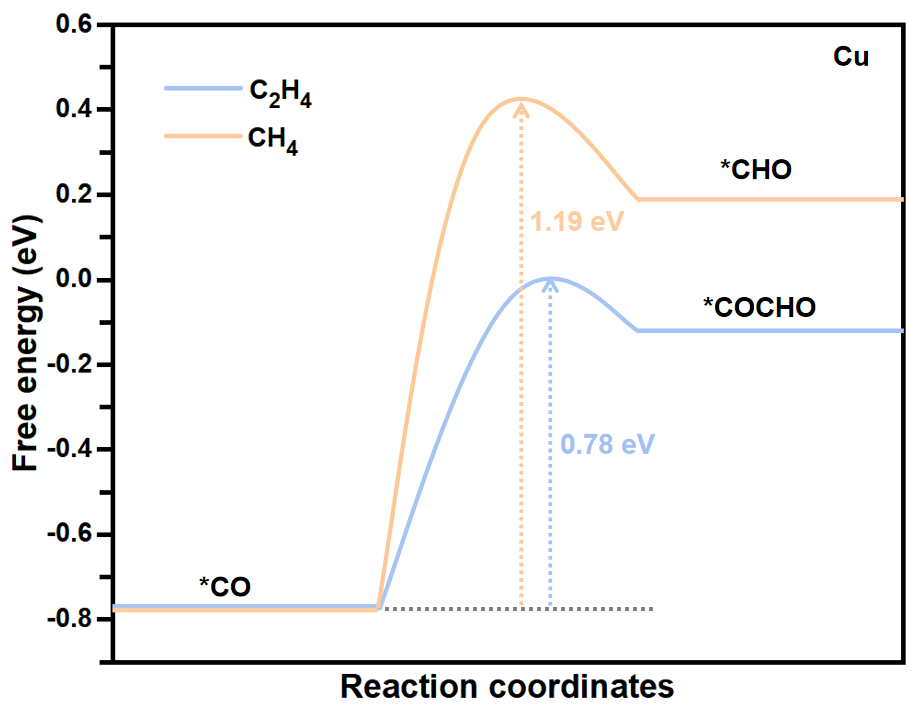


**Figure S29.** The activation energy calculations for the conversion of *CO to *CHO and *COCHO on Cu.

**Table S1.** Crystallographic data of Cu_3_Pd and Rietveld refinement data.

|  | Source |  |  |  | Laboratory X-ray | | |
| --- | --- | --- | --- | --- | --- | --- | --- |
|  | Chemical formula | | |  | Cu_3_Pd |  |  |
|  | Formula weight | | | | 297.058 |  |  |
|  | Temperature (K) | | |  | 298 |  |  |
|  | Pressure | | |  | ambient |  |  |
|  | Wavelength (Å) | | |  | 1.5406 |  |  |
|  | Crystal system | | |  | Cubic |  |  |
|  | Space group | | |  | PM-3M |  |  |
|  | (NO.) | | |  | 221 |  |  |
|  | a (Å) | | |  | 3.68574 |  |  |
|  | b (Å) | | |  | 3.68574 |  |  |
|  | c (Å) | | |  | 3.68574 |  |  |
|  | α (°) | | |  | 90 |  |  |
|  | β (°) | | |  | 90 |  |  |
|  | γ (°) | | |  | 90 |  |  |
|  | V (Å^3^) | | |  | 50.06960 |  |  |
|  | Z | | |  | 1 |  |  |
|  | R_p_ | | |  | 0.0300 |  |  |
|  | R_wp_ | | |  | 0.4550 |  |  |
| Atom | Site | | | x | y | z | occupancy |
| Pd | 1a | | | 0.0000 | 0.0000 | 0.0000 | 1.0 |
| Cu | 3c | | | 0.0000 | 0.5000 | 0.5000 | 1.0 |

**Table S2.** Elemental composition of Cu and Pd in different Cu–Pd catalysts determined by inductively coupled plasma optical emission spectrometry (ICP-OES).

| **Sample** | **Element** | **Reported concentration (mg L^-1^ )** | **Atom ratio**  **(Cu:Pd)** |
| --- | --- | --- | --- |
| Cu_3_Pd | Cu | 10.13 | 3.09:1 |
|  | Pd | 5.45 |  |
| CuPd | Cu | 14.25 | 1.15:1 |
|  | Pd | 20.54 |  |

**Table S3.** Rietveld refinement data fitting results of Cu and Pd K-edge EXAFS data of Cu-Pd alloys

| Sample | EXAFS | bond | CN | R (Å) | σ^2^ (10^-3^ Å^2^) |
| --- | --- | --- | --- | --- | --- |
| Cu_3_Pd | Cu K edge | Cu-Cu | 2.58 | 2.23 | 14.66 |
|  |  | Cu-Pd | 8.65 | 2.11 | 19.98 |
|  | Pd K edge | Pd-Pd | 2.98 | 2.01 | 12.36 |
|  |  | Pd-Cu | 8.16 | 2.36 | 6.53 |

**References**

[S1] P. E. Blöchl, Projector augmented-wave method. Phys. Rev. B **50 (1994)** 17953-17979.

[S2] K. Kresse, J. Furthmüller, Efficient iterative schemes for ab initio total-energy calculations using a plane-wave basis set. Phys. Rev. B 54 (1996) 11169-11186.

[S3] K. Kresse, J. Furthmüller, Efficiency of Ab-initio total energy calculations for metals and semiconductors using a plane-wave basis set. Phys. Rev. B 6 (1996) 15-50.

[S4] J. P. Perdew, K. Burke, M. Ernzerhof, Generalized gradient approximation made simple. Appl. Phys. Lett. 77 (1996) 3865-3868.
